# Computational modeling of attractor-based neural processes involved in the preparation of voluntary actions

**DOI:** 10.1007/s11571-023-10019-3

**Published:** 2023-10-20

**Authors:** Azadeh Hassannejad Nazir, Jeanette Hellgren Kotaleski, Hans Liljenström

**Affiliations:** 1 Department of Neuroscience, Karolinska Institutet, Solna, Sweden; 2 School of Electrical Engineering and Computer Science, Kungliga Tekniska Högskolan, Stockholm, Sweden; 3 Department of Energy and Technology, SLU, P.O. Box 7032, 75007 Uppsala, Sweden; 4 Agora for Biosystems, P.O. Box 57, 19322 Sigtuna, Sweden

**Keywords:** Intention, Volition, Attractor networks, Neurodynamic transitions, Hierarchical control, Neurocomputational modeling

## Abstract

Volition is conceived as a set of orchestrated executive functions, which can be characterized by features, such as reason-based and goal-directedness, driven by endogenous signals. The lateral prefrontal cortex (LPFC) has long been considered to be responsible for cognitive control and executive function, and its neurodynamics appears to be central to goal-directed cognition. In order to address both associative processes (i.e. reason-action and action-outcome) based on internal stimuli, it seems essential to consider the interconnectivity of LPFC and the anterior cingulate cortex (ACC). The critical placement of ACC as a hub mediates projection of afferent expectancy signals directly from brain structures associated with emotion, as well as internal signals from subcortical areas to the LPFC. Apparently, the two cortical areas LPFC and ACC play a pivotal role in the formation of voluntary behaviors. In this paper, we model the neurodynamics of these two neural structures and their interactions related to intentional control. We predict that the emergence of intention is the result of both feedback-based and competitive mechanisms among neural attractors. These mechanisms alter the dimensionalities of coexisting chaotic attractors to more stable, low dimensional manifolds as limit cycle attractors, which may result in the onset of a readiness potential (RP) in SMA, associated with a decision to act.

## Introduction

### Background

Human life is full of decisions and actions that are taken to be totally voluntary. In everyday life, our decisions could, for example, concern the choice of food to eat, or of means of transport from home to work, which is highly dependent on societal/environmental and intrinsic information. In order to be able to unravel the mysteries of conscious (free) will, it is essential to understand which and how neural structures are encoding information during intentional control.

Different views exist on the temporal and causal relationship between intentions and decisions, and to what degree intentions are conscious (see e.g. Haggard 2008, 2019; Mele 2009, 2019; Block 2021), but the difference partly depends on the context. For example, experimental situations are usually quite different from ecological ones. Here, we adhere to the view that intentions, which may be unconscious or conscious to various degree, in general precede decisions, which typically would be conscious. This view links closely to that of Freeman (1999), who based his view primarily on physiological and behavioral studies of animals.

The process of intentionality may comprise the operations of predicting, planning, and learning actions, while intentions could imply the creation and projection of alternative future states, desired, avoided, or feared. Such hypotheses could be constructed in attractor dynamics by extrapolation from past experience, controlling choices and directions of actions in the present (Freeman 1999; Liljenström, 2018).

In principle, there could be many intentions within our brain-mind system, conscious or unconscious, and possibly competing, but one (or a few) of them may lead to a decision to act. Intentions have been classified as distal, proximal, or motor intention, depending on their temporal relation to a decision to act (see e.g. Mele 2019, and Parés-Pujolràs and Haggard 2021). There could also be different causes for the emergence of intentions, for example genetic, or physiological, but sometimes also emotional and cognitive. Hence, intentions may originate in different parts of the brain, including the limbic system.

Decisions, on the other hand, are cognitive in nature and are primarily associated with higher cortical systems/processes. Hence, many animals may have intentions to do this or that, but never really make a decision to do so. Humans, too, while still largely influenced by our limbic system, may have unconscious intentions, perhaps leading to actions without decisions. However, some of our intentions could be cognitively and perhaps also consciously formed in neocortex, e.g. in prefrontal cortex (Haggard and Parés-Pujolràs, 2021). Typically, a distal intention may last for a longer period of time, and would precede a decision to act. Proximal intentions are usually quite close in time for an action. In some cases, when immediate actions are called for by environmental conditions, intentions and decisions could be more or less simultaneous.

*Intention* can be viewed as a precursor to conscious (free) will, as an “urge” or “desire” to act in a certain direction, to attain a certain goal. Voluntary movement, or more generally, behavior, is based on perception and past experience (memory), which are required for prediction of (inter)actions. *Attention* may provide information about the internal and external worlds, but intention guides our actions (Liljenström, 2011).

During the past decades, there has been a debate in the literature regarding some experiments apparently showing that free will is just an illusion. Seemingly, the brain knows, at least half a second in advance what “you” decide to do (Libet et al. 1982, 1983). Lately, this time window has been extended to incredible ten seconds, based on more recent experiments (Soon et al. 2008; Haynes 2011). The common interpretations of these studies have, however, been criticized (see e.g. Mele 2009, 2019; van Inwagen 2017), and alternative interpretations of the experiments can be given (see e.g. Liljenström, 2022).

A series of famous EEG experiments (Kornhuber and Deecke 1965; Libet et al. 1982, 1983; Keller and Heckhausen 1990; Haggard and Eimer 1999; see Libet (2004) for a review) are often quoted as evidence for free will being an illusion. The EEG *readiness potential* (RP), apparent only when averaged over a large number of trials, appears to precede the conscious will for spontaneous voluntary movements. However, in contrast to Libet type of experiments where arbitrary and purposeless choices are studied, more ecological experiments, where deliberate choices are made, no RP is observed (Maoz et al. 2019).

Voluntary actions can be considered to be endogenously self-initiated, and based on internal decisions and motivations (Brass and Haggard 2007; Haggard 2008), in contrast to involuntary movements. According to Haggard (2008), volition is associated with the concept of a goal-directed activity, based on both motivation for pursuing a goal and the instrumental knowledge for controlling the motor action. In this regard, the causal relation between the action and its outcome, as well as reason-action associations are major concerns for the study of goal-directed volitional behavior. Therefore, we may be able to take into consideration context-action and action-outcome associations in the presence of endogenous signal(s) as features of voluntary actions (Fried et al. 2017). These aspects of volitional actions can be addressed with the involvements of the hierarchically higher neural regions (Friedman and Robbins 2022). The feature of goal-directedness is strongly related to the concept of cognitive control, which is different from habitual behaviors (Petrides 2005). Cognitive control is a neural-based process developed based on the adaptive function of the brain. This process is about the maintenance of neural patterns, correlating goals and potential actions (Miller and Cohen 2001).

There are certain cortical areas believed to be central for cognitive control and attentional functions (Corbetta and Shulman 2002). The lateral prefrontal cortex (LPFC) and anterior cingulate cortex (ACC) are two key neural structures involved in bottom-up and top-down attention (Rossi et al. 2009; Aarts and Roelofs 2011). The intentional processes hierarchically influence the dynamics of internally/externally stimulated neural structures towards stability for framing decisions. Accordingly, a major focus in this work is modelling the functionalities of LPFC and ACC, as well as their interactions during intention, as the preparatory phase of volitional decision making.

Undoubtedly, many different structures are directly and indirectly involved in the exertion of voluntary actions. In addition to LPFC and ACC, areas such as the orbitofrontal cortex (OFC) and amygdala are structures related to emotional processes, which were included in our previous model of decision making (Hassannejad Nazir and Liljenström, 2015). One could also consider modelling the pre-SMA and SMA, where the RP has been detected, but for simplicity, in this work we do not consider activity in OFC, amygdala, or SMA. Instead, we confine our work to the major areas supposed to be directly involved in the emergence of the early intentional process, preparing for a decision to act, and preceding the activity in SMA. We do not consider direct motor intentions in this work.

#### Executive functions associated with voluntary action control

##### Decision making process

The decision-making process (DM) is described in a simple way by Doya (2008) in five steps: (1) presentation of external/internal stimuli, (2) predicting values of the associated potential decisions, (3) selection of an option, (4) actual outcome evaluation, and finally, (5) learning, based on the difference between the predicted and actual values of the potential options. According to Doya and others (Doya 2008; Fobbs and Mizumori 2014), maximizing the utility of the decisions is considered the main goal of all humans decision-making, where different types of contextual learning contribute to the knowledge-base for reaching a decision (Gariépy et al. 2014; Lee and Harris 2013).

##### Working memory

Working memory and attentional control are two strongly associated cognitive functions (Unsworth and Spillers 2010; Oberauer 2019; Kang et al. 2020). The commonly accepted features of working memory (temporarily holding, monitoring, and manipulating information) make this process an essential cognitive aspect of goal-directed voluntary actions. To accomplish voluntary actions, the selection of relevant stimuli and suppression of irrelevant ones is resulting from various procedural aspects of working memory affecting excitatory and inhibitory neurons at the site of termination (Baddeley 2012; Adams et al. 2018). Brodmann areas BA 9 and BA 46, which are laterally and highly connected subregions of LPFC, appear to be involved in working memory, where cytoarchitectural differences between these two areas bring about different functional aspects of working memory. The neural activity of these areas may represent different if–then rules, including reason-action and action-outcome associations.

#### Neural structures associated with volitional control

##### Lateral prefrontal cortex

In order to be able to make decisions, while being bombarded by numerous internal and external stimuli, our brains need to be equipped with functional specifications. Keeping on hold different received environmental information, loaded memories, projected interoceptive signals and goals are essential for controlling such a great deal of information. Moreover, shifting between these signals and managing the competition between them is indispensable. In this regard, the role of the most essential neural regions must be considered.

The extrinsic and intrinsic connectivity of neural parts located in the prefrontal lobe of cortex provide a broad contribution to cognitive controlling processes and executive functions. The cytoarchitecture and laminar organization of prefrontal neural areas seem to provide a basis for structure-based hierarchical information processing required for cognitive functions (Bludau et al. 2014). The executive functionality of the cortical laminar structure of LPFC was demonstrated by Pribram and others (Pribram et al 1952). The intra-cortical connectivity of LPFC serves as an early sensory stimuli processor for subsequent cognitive reasoning (Mushiake et al. 2006; Figner et al. 2010). Cognitive flexibility, working memory and inhibitory control are major aspects of executive functions involved in the intentional controlling process, including vetoing and self-control, as well as externally driven actions.

The LPFC, located in the frontal pole of the brain, has been recognized as one of the most important structures involved in cognitive processes (Tanji and Hoshi 2008). Functional neuroimaging of LPFC illustrates its contribution, not as an integrator of external information from associative sensory structures (i.e., early sensory stimuli), but as the recipient of internal afferents from other laminar cortical structures, limbic and subcortical areas. In this regard, the intrinsic connections within its different subregions, as well as its extrinsic connectivity with some other brain areas, demonstrate its versatile role in controlling goal-directed and stimulus driven behavior.

The functionality of LPFC is highly attributed to its neural organisation. Taking into account the Brodmann structural classification, LPFC consists of different subregions with different laminar organisation including Brodmann areas BA 9, 45, 46 and 47. Some studies also consider BA 10 as part of LPFC. Among these subregions, the architectonic organisation of BA 9, 10, and 46 are similar. These three areas are composed of six granular layers including well-defined layer IV (Petrides 2005), however, with different cellular distribution, bringing about different functional capacities (Petrides 2005).

##### Brodmann area 9

**Fig. 1:**
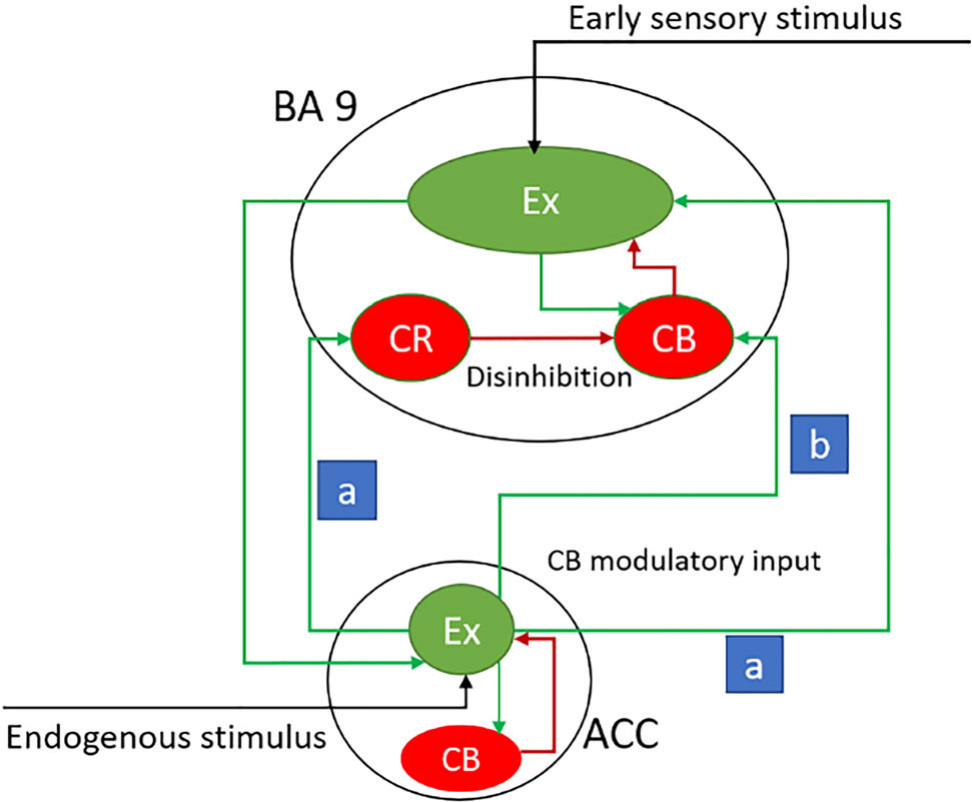
Exciting and inhibiting pathways projected from ACC to BA 9. BA 9 is composed of pyramidal neurons and various inhibitory interneurons. This area is exposed to environmental early sensory stimuli and afferent endogenous signal from ACC. The integration of these signal would bring about the unconscious selective attentional control. Providing that the internal and external stimuli are correlated, ACC excites BA 9 pyramidal neurons (Ex) through projecting directly pyramidal neurons and indirectly by exciting calretinin (CR) inhibitory interneurons resulting in disinhibition of calbindin (CB) interneurons, (i.e. pathway (**a**)). To suppress the activity of external stimulus given that the efferent signals to BA 9 are uncorrelated, the ACC excites directly the CB inhibitory neurons (i.e. pathway (**b**))

As shown in Fig. 1, BA 9 is subjected to early sensory information capable of encoding the environmental contextual information. This area also has strong connections with ACC, encoding endogenous stimuli. The high capacity of BA 9 in processing afferent endogenous signals and integrating them with environmental signals makes this structure one of the most potential neural candidates during the preparatory process of intention in the presence of endogenous signals.

This controlling process of BA 9 can be mediated through expression of calcium-binding proteins in the interneurons of this area. The abundance of calcium-binding proteins as calretinin (CR) and calbindin (CB) among the GABAergic interneurons enables BA 9 to control the environmental information considering the projected internal signal via ACC. The expression of these proteins in the interneurons activates the gating process during attentional control. Generally, CR interneurons are connected to CB interneurons, while the target of CB interneurons is excitatory (Ex) pyramidal cells. Hence, the expression of CR interneurons disinhibits the excitability of pyramidal neurons (Barinka and Druga 2010).

##### Brodmann areas 46 and 10

BA 46 and 10 are structurally similar. Both consist of six well-developed layers with the same cellular composition. However, their different cellular intensities result in distinct functionalities. BA 46 is a well-developed six laminar structure, bordered by BA 9, BA 10. The supragranular layers of this sub-region are densely organized with medium size cells (Bludau et al. 2014; Petrides 2005). This LPFC subregion is involved in executive processes such as attentional control and working memory, as well as DM (Barbey et al*.* 2013; Blakemore and Frith 2000).

BA 10 is the largest area in the frontal lobe of the brain, with a wide range of connections to various neural areas. This cortical area is known as a supramodal structure widely contributing to various cognitive functions. The adjacent regions of BA 10 are BA 9, BA 46 (as parts of LPFC), ACC and OFC, indicating the key involvement of this neural part in attentional control and DM. In addition, it has extensive connections with the pre-supplementary motor area (pre-SMA), thalamus, hypothalamus, and basal ganglia (Peng et al. 2018).

BA 10 has a lower cellular density compared to the cell density distribution of BA 46. Generally, structures with lower cellular density have greater neuropil space, and subsequently larger space for higher neural connections have been observed (Hrvoj-Mihic and Semendeferi 2019). Therefore, considerable higher neuropil fraction is likely more strongly contributing to complex functions while higher level of controlling, here referred to as hyper-controlling, compared to other LPFC subregions is required (Okuda 2007; Peng et al 2018). According to Chahine et al*.* (2015) BA 10 is involved in branching activations in a complex solving task strongly dependent on resolving goal-tree sequences. This process also increases complexity of working memory.

Considering the above-mentioned observations, Medalla and Barbas (2010) have suggested that the cellular density difference between BA 10 and BA 46 makes them play different roles in the controlling process. Therefore, BA 10 is likely to be more involved in cognitive functions with higher-order processing (Ramnani and Owen 2004).

##### Anterior cingulate cortex

Considering the unique position of ACC, this structure is connected to the cortico-cortical and cortico-limbic pathways. This structure also receives dopamine, innervated by the ventro-tegmental area encoding error related negativity. The bidirectional connections of ACC with amygdala and OFC facilitate the flow of endogenous information among these structures projecting LPFC (Bush et al. 2000; Allman et al. 2001; Palomero-Gallagher et al. 2008; Medalla and Barbas 2012; Apps et al. 2016). ACC plays an important role in modulating the neurodynamics of the LPFC through projecting its subregions during different attentional control phases. The modulatory action of this neural area is highly attributed to its laminar pattern (Palomero-Gallagher et al. 2008). Hierarchical signal transmission, determining the type of projection, is strongly related to the development of granular layer IV in cortical structures. The origin of ascending (feedforward) signals is the granular neural structure with well-developed layer IV, while the descending (feedback) signals with modulatory properties are generally projected by agranular structures which lack layer IV (Braver et al. 2001). The laminar organization of ACC, with a large densely packed layer V merged with layer VI and no layer IV, provides the structural basis of selective attention function. The controlling role of ACC during selective attention could be the result of response conflict monitoring (Shipp 2005).

ACC receives afferent signals from OFC, which has long been known as a structure involved in modulating emotional arousal (Jenison 2014; Stalnaker et al. 2015). The recorded oscillatory activity of OFC represents the expected value of the associated outcome of the afferent stimulus, which is referred to as the “expectancy signal”. Therefore, ACC is capable of projecting this expectancy signal to its connected structures. According to the hierarchical control required to manage the projected signal process, it is expected that ACC evokes recognizably different neurodynamics in each of the LPFC subregions. That is why ACC targets different neural cell types in each BA areas, resulting in the emergence of different frequencies and neural pattern behaviors.

##### Pre-supplementary motor area (pre-SMA)

The supplementary motor complex (SMC) is functionally parcellated into two areas: the supplementary motor area (SMA) and the pre-supplementary motor area (pre-SMA) (Nachev et al. 2008). The variety of observed functional activities and the outflow/inflow of information from these areas are bases for neural segregation. These two subregions of SMC, categorized as agranular structures, lack cortical layer IV. Pre-SMA has been well known as a neural structure with the function of predictive coding (Akkal et al. 2007). With regard to the neural areas adjacent to these two SMC subregions, SMA is mainly involved in movement generation, while pre-SMA provides the basis for movement initiation. Therefore, pre-SMA is considered to be strongly coupled to higher order cognitive functions (Nachev et al. 2007). Pre-SMA is characteristically well known for its oscillatory readout, and primarily the early readiness potential (RP), as a neural signature of volition. The emergence of this signal is characterized with two components (i.e. early and late RP, respectively), which supposedly are administrated by different projections from various neural structures (Seghezzi and Zapparoli 2020). The slow negative build up of the oscillatory activity consitituting the early phase of the RP, which emerges around 1500–400 ms before the movement onset, and represents the intentional preparatory process, whereas the late RP occures 400–0 ms prior to action performance (Schurger et al. 2021). The pre-SMA is exposed to afferent pathways, conveying goal-directed and motivational signals originated from different neural areas, bringing about different components of the RP. The early RP, as measured in pre-SMA, is presumed to result from input signals from LPFC subregions, while the emergence of the late RP component is the result of primary motor cortex projections (Schurger et al. 2021).

The early RP in the pre-SMA may evolve as a result of the projections of associative signals, i.e. context-action and action-outcome associations encoded by oscillatory activities of the neural attractors correlated to potential actions/goals. Hence, this cortical area mediates the required preparatory processes of DM. Subsequently, the late RP, and eventually voluntary action, would appear through bidirectional interactions between the pre-SMA and SMA, as well as some other subcortical areas, such as basal ganglia (Nachev et al. 2008).

### Focus and objectives

Although there have been many advances in the past decades in understanding the role of the brain in decision-making and volition, there are still many unanswered fundamental questions targeting the intentional control process, preparing for a decision to act. In order to shed light on these neural-mental relations, some fundamental questions should be addressed. We propose that a mathematical representation of the neurodynamics related to cognition may demonstrate transitions between different degrees of order and disorder in the brain (Freeman 2000; Århem et al., 2000). Hence, a decision could be the result of a transition from an unstable chaotic attractor to a stable limit cycle attractor in the cortical networks, which could be a hallmark of the volitional control process (Sussillo and Abbott 2009). Given these assumptions, it should be essential to examine what and how neural correlates may transform chaotic dynamics to stabilized and controlled activity patterns, in order to understand voluntary actions.

With the conceptual framework given here, we suggest the following three hypotheses, H1-3:

#### H1

Voluntary actions are the result of a reorganization of chaotic high-dimensional activity patterns to stabilized low-dimensional activity patterns.

This hypothesis is related to the question of decisions made during arbitrary and deliberate choices.

#### H2

Three LPFC subregions, BA 9, 10, and 46, as well as ACC are necessary for the intentional control process, which precedes a decision to act.

#### H3

The succession of transient-like dynamics of the brain during intentional control can be emulated by attractor networks with transitions from chaotic dynamics to stabilized low-dimensional neural activity.

These hypotheses should ideally be tested experimentally, e.g. by EEG and fMRI measurements of relevant areas during experiments on volition, but in lack of such data, we have further developed and used a phenomenological attractor based neurocomputational model, originally developed by Liljenström (1991). The output of this model can be expressed as the averaged frequency and amplitude of simulated EEG signals. Previously, the original model has been used to simulate cortical neurodynamics in functions such as perception (Liljenström, 1995; Liljenström and Wu 1995), associative memory (Liljenström, 1995; Liljenström and Hasselmo 1995) and decision making (Hassannejad Nazir and Liljenström 2015). In the following section, we describe our model in more detail, and the way it has been implemented in our modeling of the neural activity associated with intentional control.

## Theory and model

As mentioned above, the focus of this study is to scrutinize the states of attractor networks and the mechanisms underlying the reorganization process of attractor states during intentional control, as the preparatory phase of voluntary action control. In this context, we regard the brain as a nonlinear system, capable of hosting oscillatory and high-dimensional chaotic neural patterns. This prominent feature of the brain may provide a basis for deliberate decision making and adaptation. We hypothesize that a decision could be associated with a transition from a chaotic to a stabilized oscillatory brain state, and explore the possibility for how a control mechanism of chaotic neural attractors could be a new approach to the “neuroscience of volition”.

### Theory of attractor-based voluntary action control

#### Attractor-based decision-making

Simply described, decision making (DM) is about selecting an option among potential alternatives. This one-line definition can be attributed to any type of DM including habitual, self-initiated or externally-driven action selection processes. However, it can be questioned whether the implicit process in this definition is the same for all types of DM processes.

The common definition of a five-step DM, as mentioned in the Introduction, embodies a conscious control process, which is the hallmark of volitional decision making. In this regard, we may claim that “implementing successful *conscious* decisions” is thoroughly dependent on the adaptive behaviour of the brain, based on executing a set of orchestrated processes. Hence, weighing and accordingly selecting potential options could be exclusively dedicated to a volitional DM. Accordingly, what we could expect to observe in the brain before making a “conscious” decision is high-dimensional chaotic neural patterns correlated with external/internal stimuli.

Hence, in order to be able to measure the hallmarks of the neural processes, the complexity of the neural networks needs to be reduced. Consequently, the dynamics of cortical neural networks are represented by artificial low-dimensional attractor networks that can be described by coupled differential equations.

Attractors are recurrent activity patterns that the neural networks can settle to and which can help us to simulate neural functionalities associated with voluntary action controls. Memory formation, language processing, and preparatory processing of intention and DM are examples of neural functions modeled with the help of attractors (Albantakis and Deco 2011; Hutt and beim Graben 2017; Schoemann and Scherbaum 2020). The attractor neurodynamics in different neural areas determine the functionality that a cortical area is involved in (Freeman 2000). As suggested above, the attractor activity patterns of BA 9, exposed to internal and external stimuli, are supposed to behave chaotically.

#### Voluntary action control as a hierarchical DM process

**Fig. 2:**
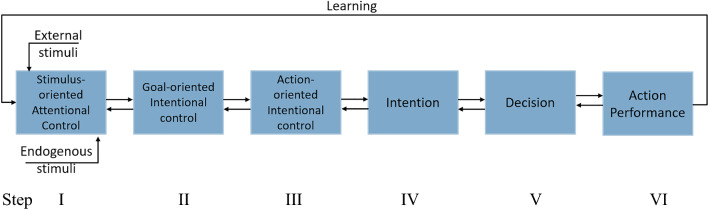
The hierarchical voluntary action control process. Efferent external and internal stimuli provide bases for the retrieval of long term memories. The first step (I) of the process is attentional control of received external and internal stimuli. The second step (II) is intentional control of goals, associated with integrated external and internal stimuli. The signal correlating to the selected goal(s) bring about the recall of the relevant potential actions. In the third step (III) potential actions are considered. The intentional control procedures comprise the initial phase of intention (IV). The fifth step (V) is making the final decision, which may lead to an action performance (VI)

The five-step DM can be associated with different subregions of the LPFC, responsible for different levels of volitional decisions. As Fig. 2 illustrates, different control procedures are exerted hierarchically to make a final conscious decision to act. In the first step (I) of this hierarchical process, there is an attentional control of received external and internal stimuli, followed by initial decisions on “goals” (Katayama et al. 2019). The steps II and III in Fig. 2 comprise the initial phase of intention, while step IV indicates the time period the agent is intending to make a specific decision (step V) to act (step VI), resulting from the previous steps. The outcome of the action will eventually be evaluated and stored in memory, for possible update of future decisions.

Every day we make various (distal and proximal) intentions with different time scales, which are also considered here: (1) the initial phase of intention (steps II and III), and (2) the process of intention, preparing for a decision to act (steps IV and V).

1. The initial phase of intention includes intentional goal control (step II) and intentional action control (step III). The time scale for this process depends on many different parameters, including optional goals and choices for action, external and internal stimuli, and loading long-term memories, etc.
2. The period of intention is associated with the required time to make a final decision. In case of distal intentions, this period may include some kind of cost–benefit evaluation of various optional choices and also consideration of consequences of the optional actions, in particular for deliberate choices. The time period is usually much shorter for proximal intentions.

The time scale for both of these phases (1 and 2) may differ dramatically depending on circumstances. If we are considering where and how to travel for our vacation, the time scale may be days or months, while if we are considering choosing/picking a food item in a grocery store, the time scale could be seconds.

The temporal scale in the first case is normally too long and complex for experimental studies, while the latter case could be studied in goal-directed volitional experiments. In Libet type of experiments, with arbitrary choices, the time scale is typically seconds.

In the present work, we consider time scales relevant to experimental circumstances, i.e. seconds, although in principle, much longer time scale could be imagined. We primarily focus on more realistic, deliberate choices, which are more related to conscious (free) will, than the simplified Libet type experiments.

In the following, we will describe our modeling of steps I–IV in the hierarchical process of voluntary action control, as described in Fig. 2. We use our model to analyze the attractor neurodynamics in different neural structures at each hierarchical level, illustrating the preparatory process before a decision to act. We refer to this process as the *intentional control* process. In attractor models of intentional control, relevant information for decision making (e.g. optional choices, goals, and memories) are settled as activity patterns, i.e. as attractors, continuously integrated in the state space of the systems considered.

### Computational model of attractor-based intentional control

#### Model of cortical interactions

We live in a world where it is almost impossible to eliminate environmental stimuli. However, in order to study the intentional control process, it seems reasonable to focus on endogenous signals. In this study, ACC has been modeled as an area transferring afferent signals from emotion-related neural structures, as well as mesolimbic dopamine to the LPFC. 

#### Feedforward and feedback control

Control is a goal-oriented process embedded broadly in the nervous system and its interactions with the environment. This process ensures the achievement of a goal by monitoring the input or output according to the type of control. Feedforward control is a linear process with the major focus on inputs, while feedback control monitors through gradual evolution of error signals in the system.

The need to correct the control process with regard to output determines the leading control system. This difference can be observed among behaviors that are driven voluntarily or triggered externally. An important question is what drives any of the controlling systems: endogenous stimuli or external/habitual stimuli. In other words, an individual may decide to follow an external stimulus, such as a red traffic light (i.e. voluntarily decides to follow an immediate external stimulus), or may habitually press the breaks after the appearance of the red light.

This example can be rephrased with a question: “What causes an individual to make either a conscious volitional action, or merely unconsciously follow an external trigger?” To address this question, we demonstrate in our model how a feedback loop would be triggered. In the following, we use our model to suggest how the ACC-LPFC interaction is capable of contributing to intentional control.

#### ACC-LPFC subregion interactions

ACC controls LPFC neurodynamics through interacting equally with all the three considered subregions of LPFC: BA 9, 10, and 46. However, targeting different neural types (excitatory or inhibitory) in each subregion leads to different functions. The hierarchical controlling process occurs through the reciprocal connections of ACC with these three subregions.

Two other prominent features of intentional control, goal-directedness and causal attribution of actions, have been modeled through two separate ACC-LPFC pathways.

**Fig. 3:**
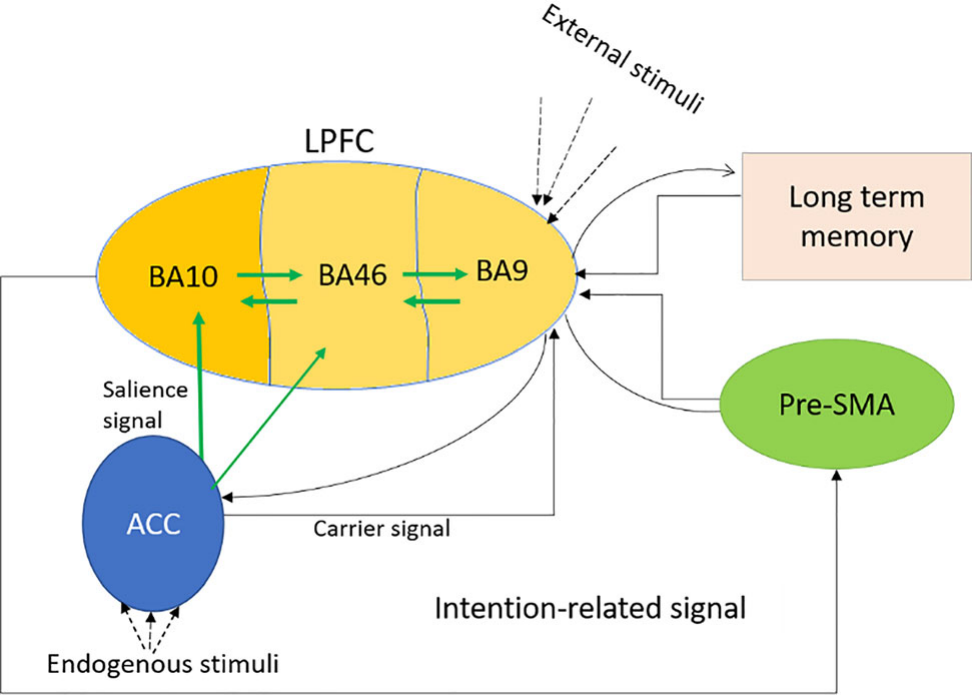
Illustration of the schematic flow of information among neural structures involved in intentional control. ACC is subject to projections from OFC, LPFC and mesolimbic dopaminergic pathway signalling expectancy signals about action-outcome (goal-directedness) contingencies. This area stimulates three subregions of LPFC: BA9, BA10 and BA46. These three areas are bidirectionally connected. ACC transfers interoceptive and emotional signals to BA9, which is also subject to environmental stimuli. The integrated signals loading into long term memory. Accordingly, the retrieved stored memories about potential actions and goals propagated to the three subregions of LPFC. The projected goal-correlating signal to BA10 receives signals correlating the internal signals projected to ACC and to BA10. Based on the measure of coupling between the projected signals to BA10, some would be enhanced and some suppressed. The enhanced signals stimulate the associated action in BA46. Here, ACC encodes BA46 based on the expectancy signals received from OFC. This brings about a competition between the potential actions. The one(s) with higher correlation would be the chosen actions signalled to the pre-SMA. BA10 also stimulates the intentional preparatory process in the pre-SMA

BA 9 is the first LPFC subregion exposed to projections from the ACC. The signal from the ACC particularly encodes endogenous information in the BA 9, while this area is also subject to environmental stimuli. The degree of correlation between the activities of externally generated cell assemblies and internally encoded neural patterns in the BA 9 determines the orientation of attention. This attentional filtering and orienting is presumably unconscious. The efferent signal projected from ACC is labeled as “carrier signal” and is illustrated in Fig. 3. The integrated signals automatically load associated long-term memories and relevant goals. The recalled long term stored goals and optional choices represent the contextual bases (environmental/internal) of retrieved potential actions, projecting two other LPFC subregions: BA 10 and 46.

#### Time–frequency based phase-amplitude coupling

Considering the fact that the projected signal from ACC and the oscillatory activity of the neural patterns in BA10 are in two different frequency bands, theta and gamma, respectively, the coupling process between signals serve a functional role in managing the goals. It could be in a way that the phase of the slower afferent signal from ACC modulates the amplitude of the oscillations with higher frequency in BA10, supposedly in a closed loop, continuously updated by the strength of coupling and frequency power. To characterize this process, the Hilbert transform of the analytic signal of the high/low pass filtered signal is computed. Accordingly, the level of coupling between the high amplitude signal and the low phase signal are measured in the model.

The retrieved goals associated with the potential choices are competing in BA10, considering the integrated internal and external stimuli. The competition could be managed by “salience signal” from ACC stimulating the excitatory layer of BA 10. In this competition, the relevant signals to the endogenous signals will be enhanced and the irrelevant ones will be suppressed. This is the second controlling process during hierarchical DM. The goal-directedness feature of the voluntary control process is supposed to be addressed in this area.

#### Phase space reconstruction

As stated above, neural patterns may behave chaotically while they are exposed to internal or external stimuli. To predict the behavior of such neural activity patterns during the intentional process, the model scrutinizes the time-structure based evolutionary changes of attractor features (i.e. the dimensionality and level of disorder), by presenting the temporal data in phase space. The reconstructed phase space characterizes the embedded fractal dimension of the attractors and its “entropy” based on Takens’ theorem (Takens 1981).

#### Phenomenological model

**Fig. 4:**
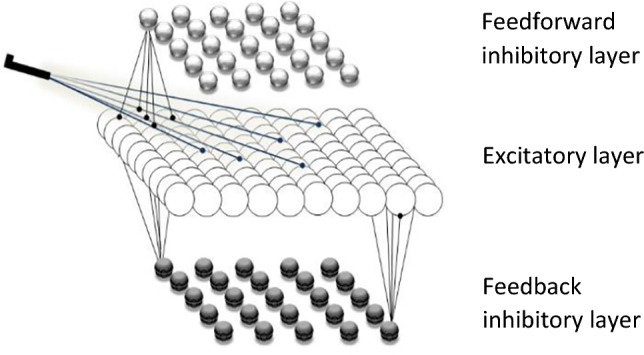
Schematic illustration of the simplified three-layered neural structures, consisting of excitatory and inhibitory neural units. The middle layer consists of 100 neural units, with one layer of 25 feed-forward inhibitory units above and 25 feedback inhibitory units in a layer below. There are extensive connections between the excitatory units, and more local connections between excitatory and inhibitory units, with no inhibitory-inhibitory connections present in the model. Afferent fibres from other areas connect only to the excitatory layer

To be able to simulate the evolution of neural activity patterns during the intentional preparation for a volitional decision, we apply a phenomenological cortical network model, originally developed by Hans Liljenström (1991), but which here has been extended and modified to mimic the structures of LPFC and ACC, respectively. The values of the parameters used in the model are listed in Table 1 in the Appendix). The schematic network structure of these two cortical areas is given in Fig. 4, where the six layers of the real structures have been lumped into three layers in the model: an excitatory layer (pyramidal cells) with one inhibitory layer (of interneurons) above and another below. An oscillatory/chaotic dynamics may result from a balance between the bidirectional connectivity of the excitatory and inhibitory layers (Liljenström, 1991; Liljenstrom and Wu 1995). The network units are non-spiking with the “mean membrane potential” as model output, stimulated by external/internal stimuli.

The cortical neurodynamics is modeled with first order differential equations, where the mean membrane potential (${u}_{i}$) of each network unit is given by Eq. 1, where *wij* is the connection (“synaptic”) strength between units *i* and *j*, *δij* is the conduction delay between units *i* and *j*, *τi* is the membrane time constant, *gi*(*ui*) is a sigmoid gain function, *I*_*i*_ is external input to unit *i*, and *ξ*(*t*) is a noise term.

1 $$d{u}_{i}/dt=-{u}_{i}/{\tau }_{i}+\sum_{j\ne i}^{N} {w}_{ij}{g}_{j}\left({u}_{j} (t-{\delta }_{ij})\right)+ {I}_{i}\left(t\right)+ \xi (t)$$

The input–output function, *g*_*i*_(*u*_*i*_) was experimentally determined by Freeman (1979) and is based on a gain parameter, *Q*_*i*_, denoting the level of arousal, or the excitability and *C* is a constant.

2 $${g}_{i}\left({u}_{i}\right)=C{Q}_{i}\left(1-\mathrm{exp}\left[-\mathrm{exp}\left({u}_{i}\right)/{Q}_{i}\right]\right)$$

To allow for learning, the connection weights are incrementally updated according to a learning rule of Hebbian type (Hebb 1949; Liljenström, 1995):

3 $$\Delta w_{ij} \, = \,\eta g_{i} \left[ {u_{i} \left( t \right)} \right]g_{j} \left[ {u_{j} \left( {t{-}\delta_{ij} } \right)} \right]\left( {w_{max} {-}w_{ij} } \right)$$

where the learning rate is denoted by *η*, and *w*_*max*_ is the maximum strength of an intrinsic synaptic connection.

This model is implemented to simulate the behavior of the two cortical areas, LPFC and ACC during the intentional preparation for a decision, preceding the activity in (pre-) SMA. As described before, the laminar organization of these two cortical areas are, respectively, granular with clearly developed layer IV and dysgranular with thin layer IV. The structural differences between these areas provide a basis for feedback projections from ACC to LPFC, encoding reward expectancy signals. The implemented model for both cortical areas is the same, but with different cytoarchitectural-related parameters. Considering the described hypothetical argument about a hierarchical decision-making process, the neurodynamics of LPFC is studied by modelling its three critical subregions, BA9, BA 10 and BA46, as well as their interaction. All these three regions belong to the same neuronal granular laminar structure. However, slight differences between their neural intensity and inhibitory neural types have been considered in modelling.

It should be noted that our model, as well as the underpinning concepts and theories, are not intended to describe Libet-type experiments, where spontaneous finger movements are correlated with a conscious “urge” to move. However, as our model clearly focuses on goal-directedness, suggesting a mechanism for intentional preparation of volitional actions, we believe our model could be applied to experiments addressing these aspects of (primarily deliberate) volition.

## Simulations and results

### Oscillation characteristics

In order to test the first hypothesis (H1) proposed in the Introduction, we investigate the time evolution of neural activity patterns in LPFC and ACC with the help of our neurocomputational model (described above) of these cortical areas and their neurodynamics. In particular, we simulate attractor network dynamics in these systems during the preparatory process of intentional control (Steps I–IV in Fig. 2) before a decision to act. The results may explain the relationship between the controlling statutes and certain features of the attractor dynamics of this process, which we study in a time, phase, and time-frequency domain. We propose that time-domain EEG-like signals generated by the model are capable of elucidating factors associated with the oscillatory behavior of the brain areas involved.

#### Time-domain signal

**Fig. 5:**
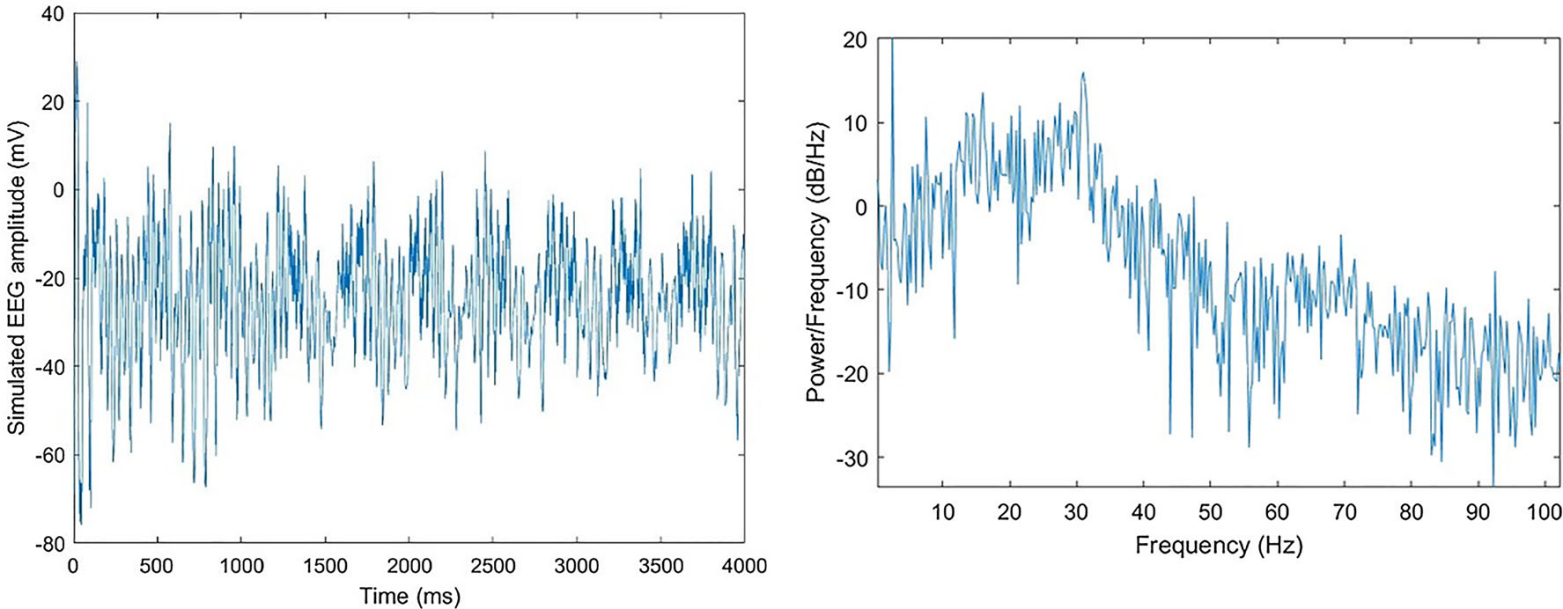
Simulated EEG-like time-series oscillatory activity of excitatory neural units in Brodmann area 9. (Left) The balanced excitatory-inhibitory oscillatory activity in BA 9. (Right) The frequency domain of the signal. Here, the dominant frequency is in theta band

The readout of the model is an EEG-like one-dimensional time series signal, representing the oscillatory neural activity pattern of a cortical structure, like LPFC and ACC. The simulated time-domain EEG signal, illustrated in Fig. 5 as a one-dimensional variable, may provide us with information about the time-dependent frequency and power spectrum of the neural oscillations. This figure presents the neural activity and the relevant frequency properties of an oscillating pattern in BA 9 related to Step I in Fig. 2. However, to have comprehensive information about the states of neural attractors in time, it is essential to analyse multivariate information, so the oscillatory activity needs to be defind in a higher dimensional space. In the following, the attractor neuodynamics are analysed in a reconstructed phase space.

#### Reconstructed phase space

In order to study the attractor neurodynamics during the intentional control process (i.e. Step II and III in Fig. 2), the emerged time-dependent signals in BA 10 and BA 46 should be reconstructed in a higher dimensional space. This high-dimensional reconstructed phase space is enriched with information about the characteristics of the nonlinear dynamics of neural attractors. The m-dimensional time delay embedding of the oscillatory signals in BA 46 is shown below (Takens 1981; Matilla-García et al. 2021).

**Fig. 6:**
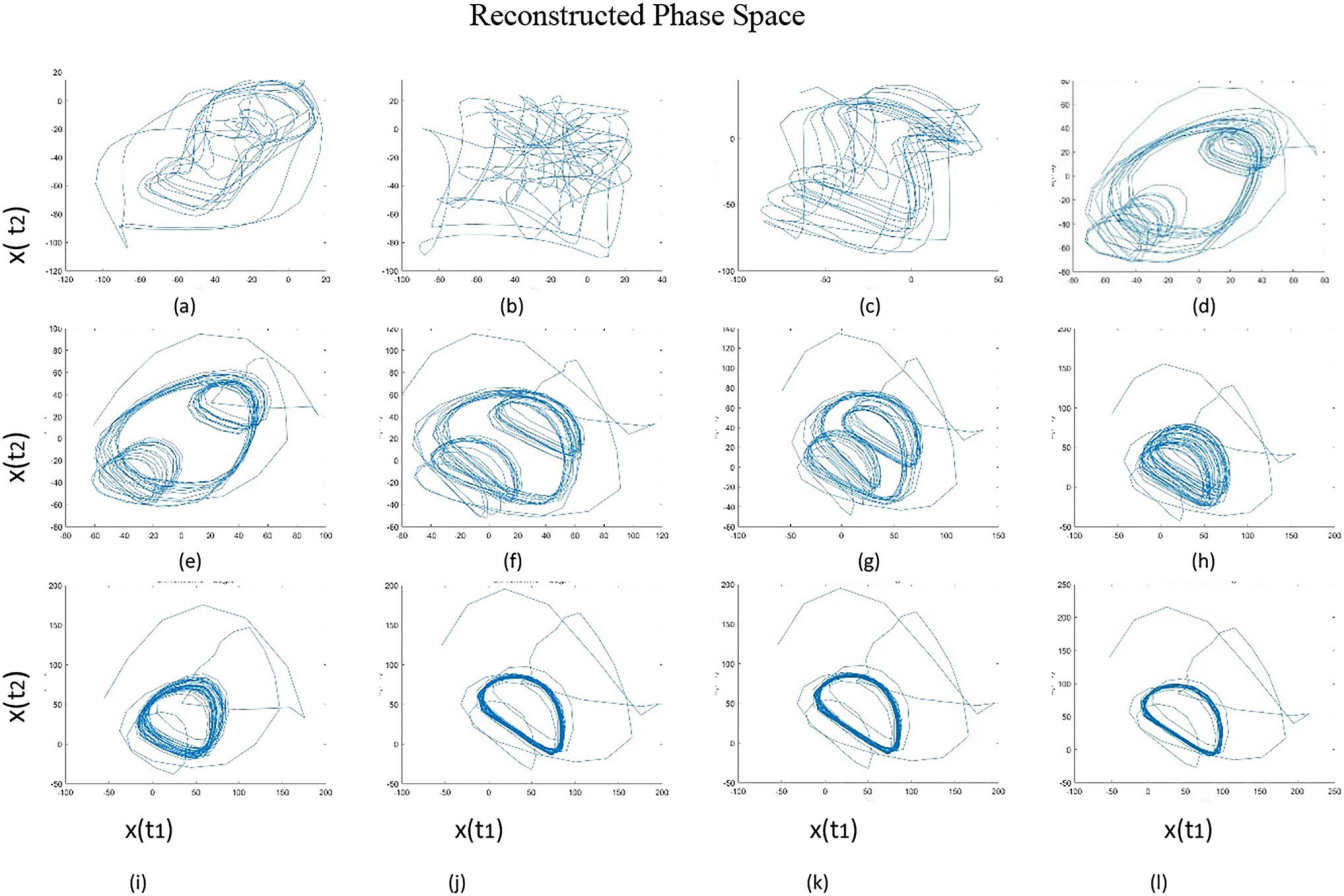
Convergence of a chaotic to a near limit cycle attractor. The illustrated patterns are the reconstructed phase space of attractors correlated to an optional choice in BA 46 during intentional control. **a** illustrates the chaotic dynamics of a neural activity pattern correlated to an option. **b**–**l** During different controlling steps the random disordered behaviour of the pattern transform into a near limit-cycle attractor

Figure 6, which is an illustration of Step III in Fig. 2, indicates the time evolution of an attractor state under interaction-dominant dynamics during the intentional control in BA 46. Here, the model explores the behavior of the attractor in twelve iterations, while this area is involved in a feedback loop with ACC (Fig. 6a–l). The number of iterations mimics the deliberating process, which is similar to the convergence process during the intentional goal process in BA 10.

Attractors have different embedding delay and surrogate dimensions in each iteration. The illustrated trajectories of dynamical states in each frame of Fig. 6 represent different levels of disorder. The changes of attractor behavior in the reconstructed phase space can be quantified with the measure of either *correlation dimension*, *Lyaponuv exponent,* or *entropy level*. In this regard, it is possible to measure the complexity of the signal in terms of such measures in the attractor fractal space.

**Fig. 7:**
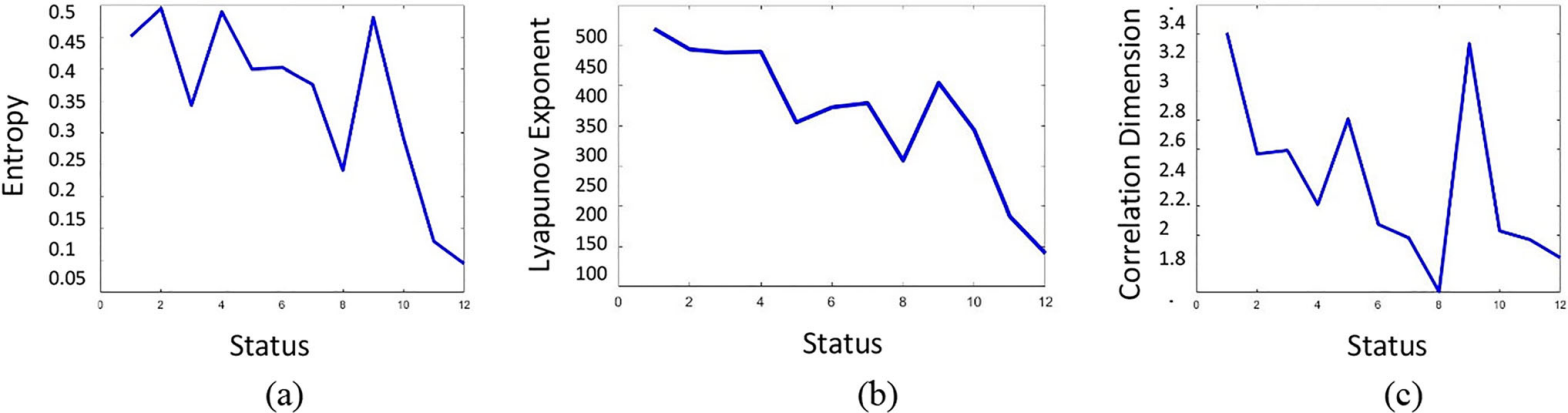
The level of disorder during the intentional control process. **a** Entropy indicates the reduction in the state of disorder of attractors. **b** The Lyapunov exponent is another measure of disorder demonstrating the stabilization process of attractors. **c** The reduction in correlation dimension of the attractor approves the reduction of energy of the system. The convergence of an activity pattern from a chaotic to a limit cycle attractor results in reduction of its dimensionality, here from 3.8 to 1.8

The observed changes in the level of disorder during convergence of a chaotic to a near limit cycle attractor in Fig. 6 are illustrated in Fig. 7. Figure 7a illustrates how the state of an attractor’s entropy, as a measure of disorder, changes during the intentional action control (Step III in Fig. 2). In general, the disorder of the attractor increases reaching a value close to zero. The same process can also be analysed by measuring the *Lyapunov exponent*, which quantifies the degree of freedom.

This measure also demonstrates the level of disorder in the system, as illustrated in Fig. 7b. The reduction of an attractor’s entropy indicates the convergence of the attractor state, from chaotic to limit-cycle. The *correlation dimension* of an attractor is the measure of complexity of the system. In Fig. 7c, this value decreases from 3.8 to 1.8, illustrating the downward trend of the attractor’s disorder.

#### Time–frequency domain

In each iteration of the simulation, reorganization of the neurodynamics from chaotic to more stabilized attractors is the result of a feedback loop between ACC and BA 46/BA 10. The characteristic structural coupling processes between ACC and the different subregions of LPFC bring about oscillatory activities in different frequency bands. An analysis of the time-series signals of each subregion in the frequency domain may determine the role of each feedback loop, making large contributions to reorganisation of neural attractors.

**Fig. 8:**
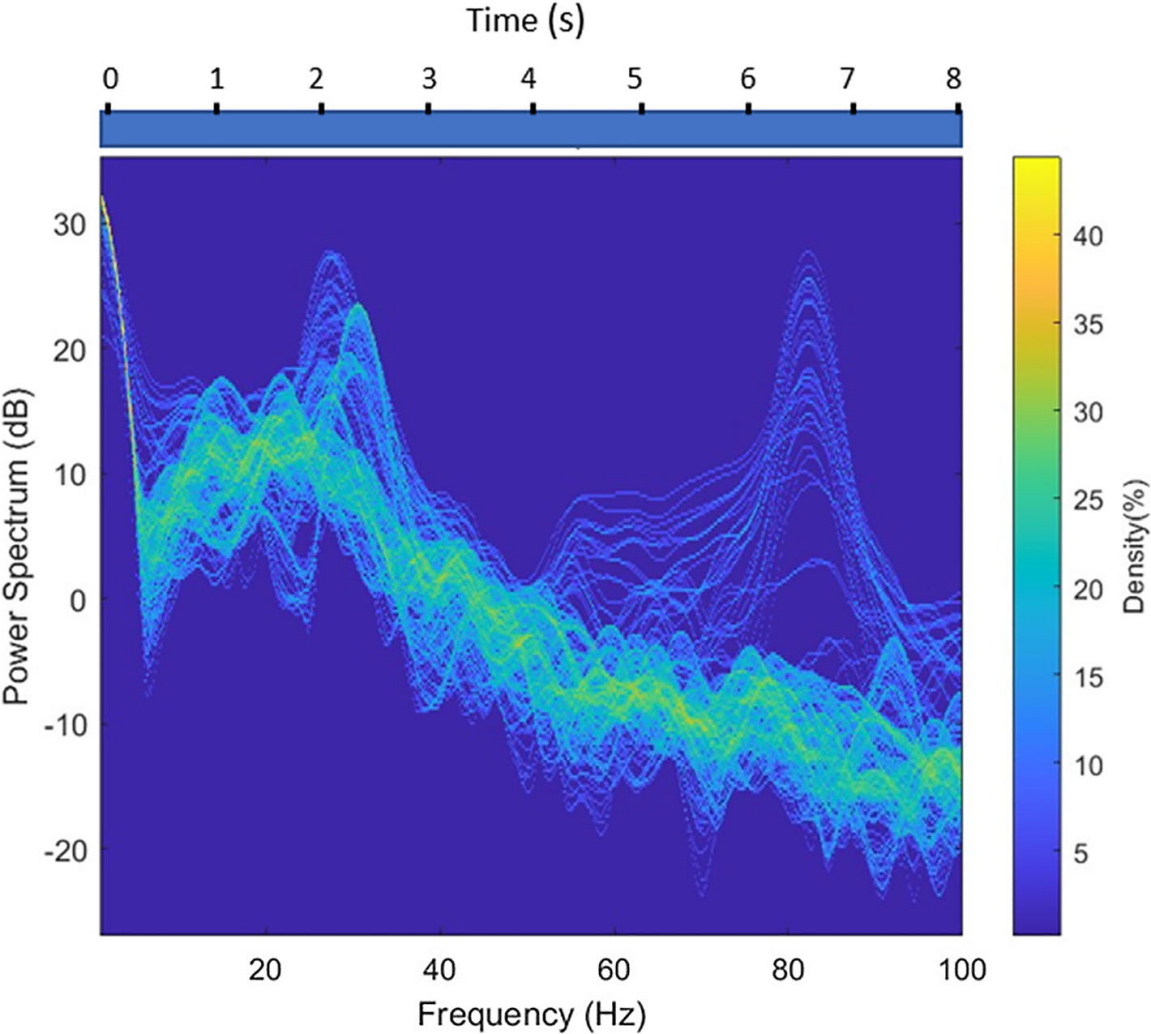
Frequency spectrogram of the goal-related signals in BA 10, which manages competition between the retrieved signals correlating associated goals to potential choices. The competition between relevant and irrelevant patterns give rise to the gamma—beta interplay. The cytoarchitecture of BA 10 (i.e. low cellular density and more neuropil spaces) provides a basis for high-level of control over goal-related patterns. This intentional goal control results in the increase of frequency bands to high beta and low gamma frequency band. The interaction between theta and gamma frequencies illustrates the stabilization process of competing relevant patterns

**Fig. 9:**
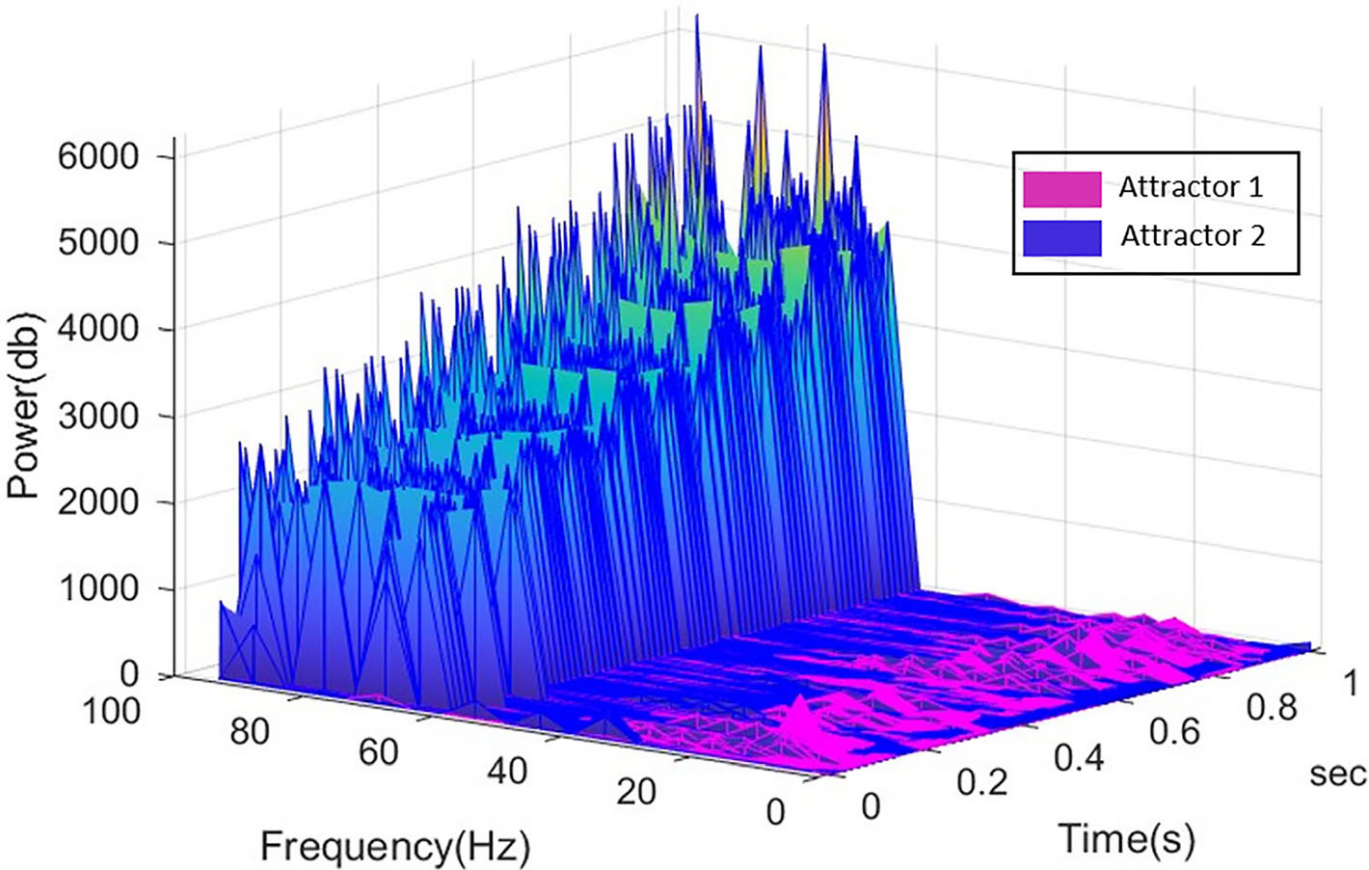
Illustration of simultaneous activities of two neural attractors in different frequency bands during intentional action control. With regard to the correlation between the afferent signals from ACC and the goal-correlated oscillations in the BA 10, the competing neural attractors undergo different alterations in their neurodynamics. The activities of relevant patterns are enhanced, reaching high-power gamma frequency, while the irrelevant ones encounter suppression. Hence, these patterns oscillate with low-power theta/beta frequency

The increase in power of frequencies in higher ranges, and the subsequent emergence of beta – gamma interplay indicates competition between patterns. This process is repeated twelve times, simulating the endogenous attention span while making decision(s) on goals (Step II in Fig. 2). This repeated intentional control process is illustrated in Fig. 8. As shown in this figure, the decrease in beta power and increase in the strength of gamma frequency band engenders a beta–gamma interplay. Here, signals with higher frequency in gamma range are coupled with the ones with slower rhythms in beta frequency band, see Fig. 9.

**Fig. 10:**
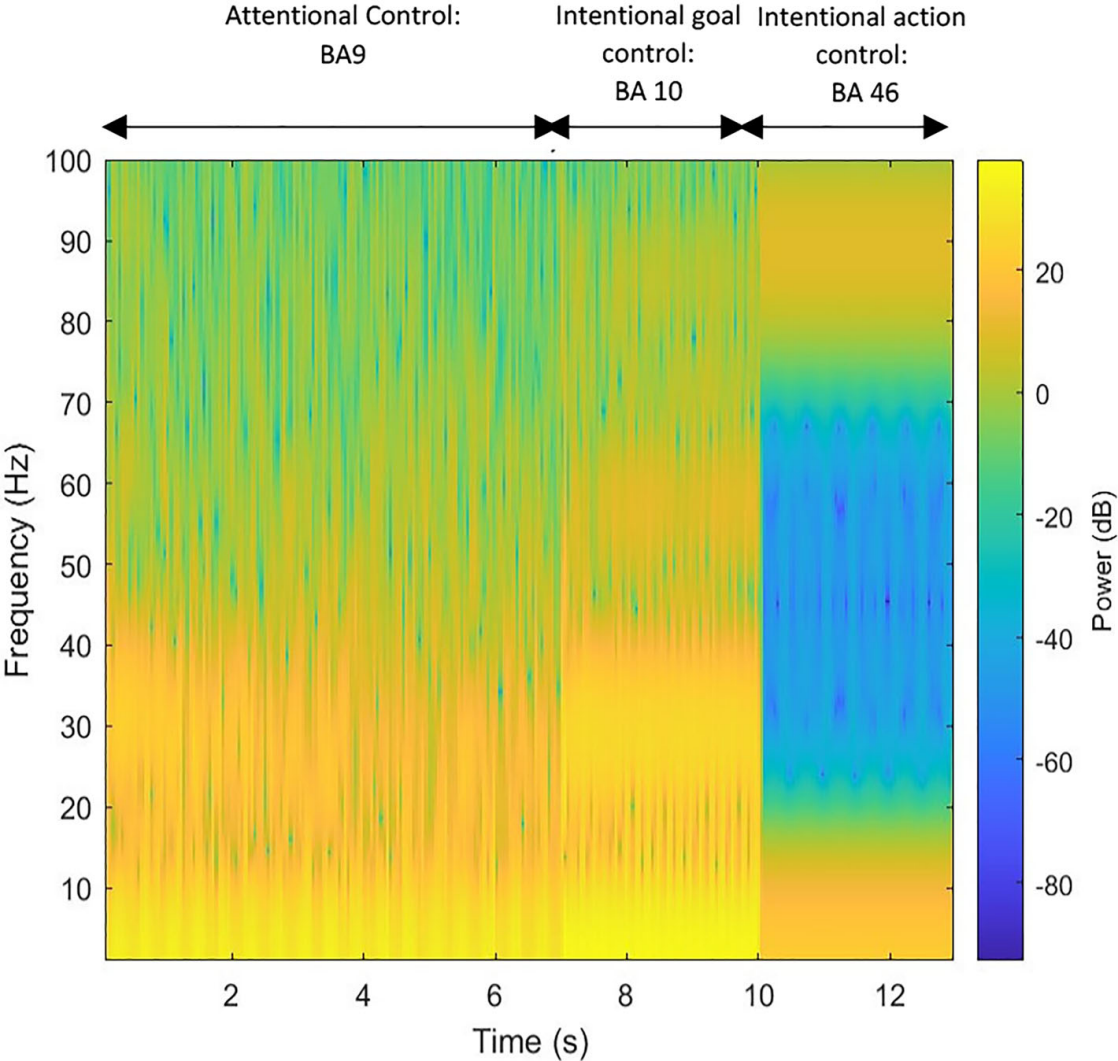
Frequency spectrum during control phases. The frequency domain of neural activity during controlling processes shifting from alpha-beta bands to gamma frequency band. The higher frequency indicates the strong competition between the potential actions. During different processes the irrelevant patterns to the dominant internal stimulus have been suppressed and the relevant ones are enhanced. The competition between the relevant patterns engenders gamma frequency

The enhanced goal-relevant patterns oscillating in gamma frequency stimulate the associated action in BA 46. Here, the model simulates the predictive coding of ACC based on the value of the expected outcomes in BA 46, generating a competition between the retrieved potential actions. This competition is the result of feedforward inhibition and stimulation of both the excitatory and feedforward inhibitory layers of BA 46. In this model, the upstream excitatory signals from ACC stimulate supragranular inhibitory neurons of BA 46 (the feedforward inhibitory layer in our model), which in turn suppress the downstream pyramidal cells (the excitatory layer in our model). Therefore, the gamma frequency oscillation is the result of a balance between excitation and inhibition, after external stimulation of layers I and II in our model. This also explains the frequency shift observed in Fig. 10. Emergence of the gamma frequency band indicates the competition between the most relevant potential actions as a result of suppression and enhancement of, respectively, irrelevant and relevant action-correlating patterns. Figure 10 illustrates, in a single frame, behavior changes of neural attractors during Steps I, II, and III.

Gamma frequency oscillations are the signature of cognitive processing. Barr and his colleagues (2009) have conducted EEG experiments recording the modulatory process of working memory in BA 46. The recorded signals in the gamma frequency band confirm what our model predicted regarding the BA 46 activity.

#### Emergence of intentional awareness

The pre-SMA is a well-known cortical structure involved in voluntary action control. LPFC projects to pre-SMA directly and indirectly through different cortical structures. Two direct pathways from LPFC subregions originate from BA 10 and BA 9. In our model, the direct pathways give rise to the emergence of early RP (Steps IV–V in Fig. 2).

**Fig. 11:**
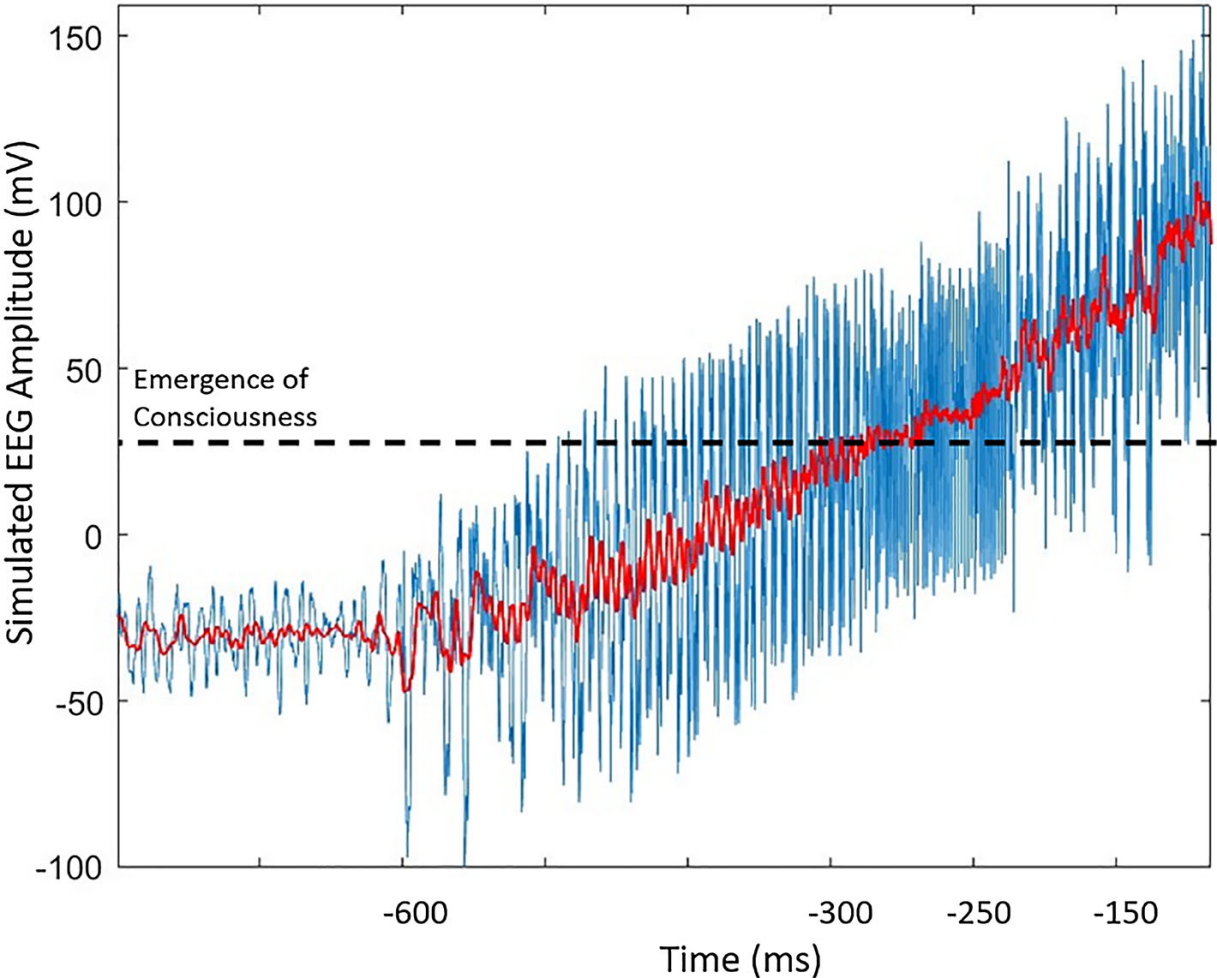
Illustration of the activity in LPFC regions BA 10 and 46, which feeds into pre-SMA. In our model, the early RP is emerged as the result of two distinct direct projections originating from BA 10 and BA9. The rising trend of early RP is predicted to be related to the enhancement of oscillatory activity of goal-related patterns (in BA10) and optional choice-related patterns (in BA 46). The broad connectivity of BA10 with subcortical areas brings about the projections of (conscious) intentional signals to this area, which afterwards would be propagated to the pre-SMA. This propagation pathway might be activated based on the specifications of oscillatory patterns in BA10. Hence, the conscious experience of our intention, related to the early RP (or other activity in pre-SMA) is dependent on BA 10 oscillatory activity patterns. The other propagation pathway from BA 9 to pre-SMA also contributes to the rising trend of the early RP leading to a decision

Oscillations originating from BA 10 propagate through laminar-based backward connections to the pre-SMA. These signals initiate the RP illustrated in Fig. 11. It is predicted that the rising trend of RP (i.e. smoothed red curve in Fig. 11) could be influenced by the enhancement of the activity in neural assemblies underlying intentional goal control. Since BA 10 is largely connected to different subcortical areas (Peng et al. 2018), this brain region is presumably involved in the conscious experience of the intentional process. This, we presume, would occur when there are synchronous oscillations (limit cycle attractors) in cortical areas. The characteristics of the oscillating patterns in the BA10 may determine the onset of the “emergence of consciousness”. However, considering consciousness as a graded process, neural patterns at different spatiotemporal scales could express different levels of complexity, corresponding to different conscious states of the individual. Therefore, any changes in the level of consciousness might be accompanied with a phase state transformation, where the complexity of the system could be defined in terms of the predictability of the neural phase space. An increase of the level of consciousness could be associated with a lower degree of neural pattern complexity, and consequently a higher predictability of the attractor behavior.

Crossing some threshold (which may vary according to the state of arousal), the neural structure(s) involved may stimulate BA 10 assemblies. This threshold crossing could correspond to a transition from an unconscious to a conscious state of mind, i.e. reflecting an increasing awareness of the intention leading to a decision to act. The decision itself would have to be conscious. The emergence of a sense of agency for an individual on the verge of making a voluntary decision is contingent on such a transition.

From a dynamical point of view, the neural entrainment by an endogenous signal in the iterative controlling process described above, could be related to a “chaotic crisis” (Grebogi et al. 1983), although possibly at a longer time scale. The stabilization of attractors can be represented with the phase trajectory controlled as a result of increasing the level of inhibition. The increase of inhibitory effects in our model is the result of the emergence of lower levels of graded consciousness into the iterative controlling process. The increase of consciousness, whereby the inhibitory effect is facilitated, is assumed to be highly correlated with the oscillatory properties during the time span considered here.

The second direct pathway originated from BA 9 cause further incremental trend of preparatory process of intention. The competitive process between the optional choices (Steps III-IV) leads to a final decision to act in SMA.

## Discussion

### Assumptions

In this paper, we have proposed three hypotheses to study the neural correlates and mechanisms underlying the intentional control process leading to a (conscious) decision to act. Here, it is suggested that characterizing the neural patterns as attractors could be an appropriate approach for unraveling this type of intentional process. Taken into consideration this assumption, we have developed an attractor-based neurocomputational model, and investigated the behavioral changes of neural activity patterns during the intentional control process, in preparation for a decision to act. With our model, we have investigated the time evolution of different attractor states. We have demonstrated that the emergence of intention is dependent on the hierarchical control of external and internal information, as well as on retrieved associated goals and actions. This hierarchical organization provides a basis for recursion to key components of the control process, thereby underpinning reinforcement contingencies at different levels. Moreover, the states of the neural attractors involved in this hierarchical step-model are controlled by feedback loops at each step. The relatively homogenous cytoarchitecture of the LPFC subregions (i.e. BA9, BA 10 and BA46), which are functionally segregated, is able to structure the hierarchical control process.

The main idea behind our modeling is based on the recorded flow of information between LPFC subregions, as well as LPFC and ACC interactions during the cognitive control process (Medalla and Barbas 2010). The structure-based functional connectivity between LPFC subregions, as well as between ACC and pre-SMA, has been guiding in developing the hypothetical ideas about various components of our model. The interactions between LPFC subregions as fairly similar granular structures have inspired a hierarchical controlling process of intention. Moreover, the critical location of BA 10, broadly receiving afferent signals from subcortical areas, and also its strong connectivity with pre-SMA has directed us to consider this connectivity to be a pathway to an increasing awareness of the intentional process towards a decision to act.

### Challenges in modeling

The main focus of this paper has been to explore possible neural mechanisms underlying intentional control. As discussed in the Introduction, certain psychophysical evidence appears to dispute the existence of free will. This conclusion is based on the observations of neural activities which correspond to the outcome of a decision (D-moment) *before* the advent of a sense of agency (W-moment) in making that decision (Libet et al. 1983; Soon et al. 2008).

By developing a computational model of the neurodynamics of brain areas associated with intentional control, we can suggest neural mechanisms underlying the observations in experiments on volition. Our results, including the demonstration of neural state transitions, suggest an explanation for those experimental observations, which we believe are just part of what happens during intentional control. Therefore, we argue that available experimental observations cannot, by themselves, be taken as evidence against free will. However, there is still a lack of relevant data for validating our model, which constitutes the main problem for this type of modeling. Ideally, simulation results such as ours, mimicking EEG/MEG signals, should be compared with experimental data collected in a similar volitional decision-making paradigm. We hope to be able to validate our model with such data in future work.

The objective of our study also came with challenges in interpreting the relevant experimental results and developing a neurodynamic model of voluntary-based actions. In order to be able to unravel the neural mechanisms involved, it was essential to consider the fundamental differences between *deliberate* and *arbitrary* choice (Maoz et al. 2019), while allegedly the initial neural states and the outcome action in both situations may be similar. An important feature that reveals the need for different mechanisms in these two situations is that different stimuli could drive these two processes. Another distinguished feature is that immediate external triggers do not necessarily initiate a decision-making process, as was described in the Introduction, while an internally triggered action would.

In this regard, taking into consideration the irregular behaviour of brain neurodynamics, the central question would be how an endogenously triggered decision-based process could result in a convergence of a chaotic neural pattern to a stabilized oscillatory one. It is a central challenge to understand the mechanisms involved in this process, studying the neural assembly states in continuous time, as well as the measure of disorder in each state.

The problems we experienced in our modeling efforts were mainly related to recognizing and designing the structure-based neural mechanisms behind reason-action and action-outcome contingencies, specifically, in the *presence of endogenous triggers*. The main challenges we faced were modeling of (*i*) the intra-LPFC pathways (i.e. the connectivity between the LPFC subregions) and (*ii*) the feedback loops between ACC and the LPFC subregions. It is noteworthy that the presence of an immediate external trigger does not necessarily lead to a deliberate action. Individuals might avoid following environmental triggers voluntarily. Therefore, in our model, external stimuli were solely counted as contextual information. However, another challenge is to unravel what initiates the voluntary pathway even in the presence of immediate external stimuli. This question raises a great challenge in modelling of conscious and unconscious volitional decisions.

The neuroimaging techniques have not yet developed enough to be able to get detailed access to neural activities in all brain areas relevant to volition (or any other cognitive function). It also seems impossible, in some cases, to distinguish the order of neural activation in terms of time. Although the observations seem to be correct, inferring based on incomplete data might lead to incorrect conclusions. Under these circumstances, the development of computational models improve our incomplete interpretations about brain functionality arising from the shortcomings of technology. We believe our neurocomputational model has been able to suggest plausible aspects of an intentional control process, preceding a decision to act. The model results presented here can, for example, explain the very early (10 s) “unconscious brain activity” in frontal cortex before the subject’s decision reached awareness, as observed by Soon et al. (2008). Indeed, this kind of modelling may contribute to a new perspective on the problem of volition and free will.

### Control pathways targeting contingencies

With regard to the features of intentional control, in our model, possible neural mechanisms underlying the intentional process leading to volitional decisions are based on inspecting the reason-action, as well as action-outcome associations. Therefore, the control pathways are based on stimulating the associative based signals. The endogenous and exogenous signals are considered to be reasons (motivations) behind retrieval of long-term stored potential goals and associated actions. Based on the evoked reason-action/goal contingencies, internal stimuli subsequently address goal-directedness feature of intentional control, by bringing about highly probable goals satisfying potential actions. In these two control processes, attractors corresponding to the actions and goals undergo changes towards either stable oscillations, or more chaotic behavior.

Simulating the neural mechanisms of the intentional control process was developed mainly based on a hierarchical recollection of long-term stored contingencies (i.e. reason-action and action-outcome contingencies). The effects of processing contingencies on attractor neurodynamics have been observed and analyzed from different aspects, which is discussed more thoroughly below.

### Attractor (in)stability associated with reinforcement contingencies

One of the contextual influences of hierarchical control is based on the level of disorder of the neural attractors. In the first step of this hierarchy, controlling exposure to endogenous (signalled from ACC) and external stimuli in BA 9 gives rise to the retrieval of potential actions and associated goals. In our model, the integrated endogenous-exogenous signals are considered to be motivation(s) behind retrieved actions. Loading of long-term stored reason-action and action-outcome associations result in changes of stored attractor disorder. This could be observed by measuring the dimensionality and/or the entropy of the attractors.

Measuring the entropy of an attractor as a neural signature of chaos in the system have provided us with information about the stability of the attractor. In the way of recollection of contingencies, integrated internal–external signal integration, as well as the strength of exposed signals, some external signals have been removed and some have been strengthened. In this regard, many related and unrelated associated actions and goals have been provoked. This process is considered to be a kind of attentional process which occurs unconsciously.

The disorder state of a neural pattern correlated to one of the potential actions has also been analysed in Fig. 6. With regards to the characteristics of any feedback loop, the enhancement of oscillatory patterns correlated to the endogenous pattern and suppression of irrelevant ones brings about stability. This process explains the mechanism controlling the convergence of an attractor in phase space, from a chaotic state to a limit-cycle one. This transformation is the result of a decrease of the disorder of the attractors, as illustrated in Fig. 6.

### Frequency changes associated with reinforcement contingencies

The general downward trend of the measures of entropy and Lyapunov exponent in the different states of feedback control, demonstrate convergence of chaotic attractors state into stability. These changes are accompanied by the changes in frequency spectrum. The gradual frequency shift from high-power alpha–beta frequency bands to high-power gamma frequency indicate a feedback loop control of potential actions. Figure 8 demonstrates the frequency shift from high-power beta to gamma band. The signal(s) in the gamma frequency band with higher power is (are) the ones intended to be selected endogenously.

### Externally triggered actions vs. (un)conscious self-initiated actions

The overall objective of this paper can be summarized in the question: “What causes an action, exerted either endogenously or exogenously?” In contrast to externally triggered actions, as was described above, the neural mechanism underlying a volitional decision is based on a closed-loop control system. An externally stimulated action is controlled by a feedforward process, while an endogenously controlled process is feedback-based.

Considering the simultaneous presence of external and internal stimuli, we suggest that the strength of these stimuli, the ratio of their powers, as well as the correlation between them, play a key role in activating any of these loops. In our model, the signal driving the feedforward or feedback loops originates in BA 9. With regard to the bidirectional interaction between this LPFC subregion and ACC, the characteristics of the integrated internal–external signals in the BA 9 may determine the pathway driving the chosen action. It could either initiate the feedback loops between ACC and the other LPFC subregions (i.e. *deliberate* pathway), or prohibit ACC propagations to other areas (i.e. *arbitrary* pathway). While an experimental study on the neural mechanisms underlying deliberate and arbitrary actions seems to show that no RP appears for deliberate actions (Maoz et al. 2019), further studies are required to unravel the significance of this.

In our model, the threshold for the emergence of conscious intention is associated with the synaptic strength of the projected attractors in the pre-SMA. Provided that the endogenous signal from ACC propagates through the LPFC subregions, initiating the deliberate pathway, the strength of oscillations in BA10 determines whether the volitional action would be taken consciously or unconsciously. We suggest that this process might be threshold-based. Therefore, voluntary exertion of the body to make an action is normally accompanied by a sense of agency.

### Precedence of the recorded D-moment to W-moment

Based on the neurocomputational modeling described above, we may be able to explain the order of D-moment and W-moment (Libet et al. 1982) during conscious volitional action. As was described in Sect. 2, BA 10 is the gate for driving the intentional control process through propagating a signal projected by subcortical structures. Hence, the efferent “conscious” signal to the BA 10 would stimulate the pre-SMA, resulting in the emergence of the preparatory process of intention. Simultaneously, oscillating signals in the BA 10, corresponding to goal competition, would propagate through BA 46, stimulating the associated actions. The retrieved potential associated actions in BA 46 are presumably exposed to ACC projections. The stimulation of excitatory/feedforward inhibitory correlating neural units in the BA46 brings about the emergence of signals propagated through BA 9 to pre-SMA, leading to the final decision(s).

The signals representing competing goals and actions in BA10 and BA46, respectively, are supposed to propagate through two pathways (i.e. BA10 to pre-SMA and BA10 to BA 46-BA9-preSMA) with some time delay. This interlude explains the reason behind the precedence of the recorded D-moment to W-moment, while the action would be made voluntarily.

## Conclusion

In this paper, we have described our neurocomputational attractor based model, which has been applied to investigate the neural mechanisms underlying intentional control. We have also addressed differences between deliberate and arbitrary actions, and described possible neural pathways for deliberate choices. Our research demonstrates that in order to be able to describe the cognitive process of intention and volition, it is necessary to understand changes in the neurodynamic patterns involved. The convergence of chaotic to (near) limit cycle attractors, as well as the observed frequency transition from beta to gamma oscillations, indicate the importance of the key role of feedback pathways in the intentional preparation of voluntary actions. Our model of the underlying neural mechanism of intentional control demonstrates how the feedback-based connectivity between ACC and different LPFC subregions engender a hierarchical intentional control process.

We conclude that current experimental observations can only explain a part of the process of voluntary actions, but not necessarily determine on the existence of free will. By developing a neurocomputational model, we have been able to provide a more comprehensive look at this process, which could lead to further experimental predictions on how to pinpoint brain processes important for our experience of conscious (free) will.

## Sensitivity analysis of the model

The neurodynamics of the lateral prefrontal cortex (LPFC)'s sub-regions (i.e. BA9, BA 46 and BA10) and the anterior cingulate cortex (ACC) are dependent on structural and dynamics related parameters listed in Table 1. The assigned values to these parameters are based on physiological measurements.

**Table 1:** The neurodynamics of the three-layered network in each of the two modelled structures, LPFC and ACC is dependent on the variability of structural and dynamics related parameter values. The assigned values are biologically plausible and based on experimental measurements

$\mathrm{N}$	Total number of network units in each of the two structures	150
${\mathrm{N}}_{\mathrm{ex}}$	Number of excitatory neurons	100
${\mathrm{N}}_{\mathrm{in}}$	Number of inhibitory neurons in each layer	25
${\uptau }_{\mathrm{I}}$	Decay time constant for feedforward inhibitory units	70 ms
${\uptau }_{\mathrm{II}}$	Decay time constant for excitatory units	7 ms
${\uptau }_{\mathrm{III}}$	Decay time constant for feedback inhibitory units	10 ms
${\uplambda }_{\mathrm{ex}-\mathrm{ex}}$	Space constant for excitatory -excitatory connections	5 mm
${\uplambda }_{\mathrm{ex}-\mathrm{in}}$	Space constant for excitatory -inhibitory connections	5 mm
$\mathrm{d}$	Distance between nearest neighbors	1 mm
${\mathrm{v}}_{\mathrm{ex}}$	Signal velocity in excitatory-excitatory connection	0.5
$\mathrm{Q}$	Gain parameter in the output function of Eq. (6)	12
${\mathrm{t}}_{\mathrm{syn}}$	Synaptic delays at all connections	0.8 ms

To determine the sensitivity of the model behavior to the inputs, we have analysed the variations of the output through a change of each parameter value by 20%. The model is robust to changes for a range of ± 20% of all the listed values in Table 1. It should be considered that the stability of the system in terms of correlation dimension will be defined in a range. So that, the correlation dimension of phase spaces in the range of 1–2 are considered stable. The stability of the attractors has been measured as a value in the mentioned stability range. However, the closer the changes were to 20%, the closer the correlation dimension was to the limit of stability (i.e. approximately 1.8). Interestingly, we also observed that as the changes get closer to 20%, the oscillatory power in gamma frequency decreases. However the entropy of the attractors have been measured around 0%. The stability of the system, considering the correlation dimension and entropy values, will be disturbed when changes exceed + 20% of the initial values. We also observed that the rate of frequency shift decreases when the changes reach ± 20% of the initial values. In addition, the number of units is not considered to be a parameter influencing the model’s behavior to a larger extent. However, we tested the model for twice the number of units listed in Table 1. The obtained results are similar to the results presented in the “Simulations and Results” section.

## References

[CR1] Aarts E, Roelofs A (2011) Attentional control in anterior cingulate cortex based on probabilistic cueing. J Cogn Neurosci 23(3):716–727. 10.1162/jocn.2010.21435. (**Epub 2010 Feb 10 PMID: 20146601**) 20146601 10.1162/jocn.2010.21435

[CR2] Adams EJ, Nguyen AT, Cowan N (2018) Theories of working memory: differences in definition, degree of modularity, role of attention, and purpose. Lang Speech Hear Serv Sch 49(3):340–355. 10.1044/2018_LSHSS-17-0114 29978205 10.1044/2018_LSHSS-17-0114 PMC6105130

[CR3] Akkal D, Dum RP, Strick PL (2007) Supplementary motor area and presupplementary motor area: targets of basal ganglia and cerebellar output. J Neurosci 27(40):10659–10673. 10.1523/JNEUROSCI.3134-07.2007.PMID:17913900;PMCID:PMC6672811 17913900 10.1523/JNEUROSCI.3134-07.2007 PMC6672811

[CR4] Albantakis L, Deco G (2011) Changes of mind in an attractor network of decision-making. PLoS Comput Biol 7(6):e1002086. 10.1371/journal.pcbi.1002086 21731482 10.1371/journal.pcbi.1002086 PMC3121686

[CR5] Allman JM, Hakeem A, Erwin JM, Nimchinsky E, Hof P (2001) The anterior cingulate cortex. the evolution of an interface between emotion and cognition. Ann N Y Acad Sci 935:107–117 (**PMID: 11411161**) 11411161

[CR6] Apps MA, Rushworth MF, Chang SW (2016) The anterior cingulate gyrus and social cognition: tracking the motivation of others. Neuron 90(4):692–707. 10.1016/j.neuron.2016.04.018.PMID:27196973;PMCID:PMC4885021 27196973 10.1016/j.neuron.2016.04.018 PMC4885021

[CR7] Århem P, Blomberg C, Liljenström H (eds) (2000) DisorderVersus order in brain function –essays in theoretical neurobiology, London: World Scientific Publ. Co

[CR8] Baddeley A (2012) Working memory: theories, models, and controversies. Annu Rev Psychol 63(1):1–29. 10.1146/annurev-psych-120710-100422 21961947 10.1146/annurev-psych-120710-100422

[CR9] Barbas H, Wang J, Joyce MKP, García-Cabezas M (2018) Pathway mechanism for excitatory and inhibitory control in working memory. J Neurophysiol 120(5):2659–2678. 10.1152/jn.00936.2017 30256740 10.1152/jn.00936.2017 PMC6295541

[CR10] Barbey AK, Koenigs M, Grafman J (2013) Dorsolateral prefrontal contributions to human working memory. Cortex 49(5):1195–1205. 10.1016/j.cortex.2012.05.022 22789779 10.1016/j.cortex.2012.05.022 PMC3495093

[CR11] Barinka F, Druga R (2010) Calretinin expression in the mammalian neocortex: a review. Physiol Res 59(5):665–677. 10.33549/physiolres.931930. (**Epub 2010 Apr 20 PMID: 20406030**) 20406030 10.33549/physiolres.931930

[CR12] Barr MS, Farzan F, Rusjan PM, Chen R, Fitzgerald PB, Daskalakis ZJ (2009) Potentiation of gamma oscillatory activity through repetitive transcranial magnetic stimulation of the dorsolateral prefrontal cortex. Neuropsychopharmacology 34(11):2359–2367. 10.1038/npp.2009.79. (**Epub 2009 Jul 15 PMID: 19606086**) 19606086 10.1038/npp.2009.79

[CR13] Blakemore SJ, Frith CD (2000) Functional neuroimaging studies of schizophrenia, (eds.): Mazziotta JC, Toga AW, Frackowiak RSJ, Brain mapping: the disorders, Academic Press, pp 523–544 10.1016/B978-012481460-8/50024-X.

[CR14] Block N (2021) Do conscious decisions cause physical actions? In: (W. Sinnott-Armstrong and U. Maoz, eds.), Free Will: philosophers and neuroscientists in conversation*.* New York: Oxford University Press

[CR15] Bludau S, Eickhoff SB, Mohlberg H, Caspers S, Laird AR, Fox PT, Schleicher A, Zilles K, Amunts K (2014) Cytoarchitecture, probability maps and functions of the human frontal pole. Neuroimage 93:260–275. 10.1016/j.neuroimage.2013.05.052 23702412 10.1016/j.neuroimage.2013.05.052 PMC5325035

[CR16] Brass M, Haggard P (2007) To Do or not to do: the neural signature of self-control. J Neurosci 27(34):9141–9145. 10.1523/jneurosci.0924-07.2007 17715350 10.1523/JNEUROSCI.0924-07.2007 PMC6672190

[CR17] Braver TS, Barch DM, Gray JR, Molfese DL, Snyder A (2001) Anterior cingulate cortex and response conflict: effects of frequency, inhibition and errors. Cereb Cortex 11(9):825–836. 10.1093/cercor/11.9.825. (**PMID: 11532888**) 11532888 10.1093/cercor/11.9.825

[CR18] Bush G, Luu P, Posner MI (2000) Cognitive and emotional influences in anterior cingulate cortex. Trends Cogn Sci 4(6):215–222. 10.1016/s1364-6613(00)01483-2. (**PMID: 10827444**) 10827444 10.1016/s1364-6613(00)01483-2

[CR19] Chahine G, Diekhof EK, Tinnermann A, Gruber O (2015) On the role of the anterior prefrontal cortex in cognitive “branching”: an fMRI study. Neuropsychologia 77:421–429. 10.1016/j.neuropsychologia.2015.08.018. (**Epub 2015 Aug 20 PMID: 26300386**) 26300386 10.1016/j.neuropsychologia.2015.08.018

[CR20] Corbetta M, Shulman GL (2002) Control of goal-directed and stimulus-driven attention in the brain. Nat Rev Neurosci 3(3):201–215. 10.1038/nrn755 11994752 10.1038/nrn755

[CR21] Doya K (2008) Modulators of decision making. Nat Neurosci 11:410–416 18368048 10.1038/nn2077

[CR22] Figner B, Knoch D, Johnson E et al (2010) Lateral prefrontal cortex and self-control in intertemporal choice. Nat Neurosci 13:538–539. 10.1038/nn.2516 20348919 10.1038/nn.2516

[CR23] Fobbs WC, Mizumori SJ (2014) Cost-benefit decision circuitry: proposed modulatory role for acetylcholine. Prog Mol Biol Transl Sci 122:233–261. 10.1016/B978-0-12-420170-5.00009-X. (**PMID: 24484704**) 24484704 10.1016/B978-0-12-420170-5.00009-X

[CR24] Freeman WJ (1979) Nonlinear gain mediating cortical stimulus-response relations. Biol Cybern 33:237–247 497266 10.1007/BF00337412

[CR25] Freeman WJ (1999) Consciousness, intentionality and causality. J Conscious Stud 6(11–12):143–172

[CR26] Freeman WJ (2000) Neurodynamics: an exploration in mesoscopic brain dynamics. Springer, London

[CR27] Fried I, Haggard P, He BJ, Schurger A (2017) Volition and action in the human brain: processes, pathologies, and reasons. J Neurosci 37(45):10842–10847. 10.1523/jneurosci.2584-17.2017 29118213 10.1523/JNEUROSCI.2584-17.2017 PMC5678016

[CR28] Friedman NP, Robbins TW (2022) The role of prefrontal cortex in cognitive control and executive function. Neuropsychopharmacol 47:72–89. 10.1038/s41386-021-01132-0 10.1038/s41386-021-01132-0 PMC8617292 34408280

[CR29] García-Cabezas MÁ, Zikopoulos B, Barbas H (2019) The Structural Model: a theory linking connections, plasticity, pathology, development and evolution of the cerebral cortex. Brain Struct Funct 224(3):985–1008. 10.1007/s00429-019-01841-9 30739157 10.1007/s00429-019-01841-9 PMC6500485

[CR30] Gariépy JF, Watson KK, Du E, Xie DL, Erb J, Amasino D, Platt ML (2014) Social learning in humans and other animals. Front Neurosci 8:58. 10.3389/fnins.2014.00058.PMID:24765063;PMCID:PMC3982061 24765063 10.3389/fnins.2014.00058 PMC3982061

[CR31] Grebogi C, Ott E, Yorke JA (1983) Crises, sudden changes in chaotic attractors, and transient chaos. Physica D Nonlinear Phenomena Elsevier BV 7(1–3):181–200. 10.1016/0167-2789(83)90126-4.ISSN0167-2789

[CR32] Groen Y, Wijers AA, Mulder LJM, Minderaa RB, Althaus M (2007) Physiological correlates of learning by performance feedback in children: a study of EEG event-related potentials and evoked heart rate. Biol Psychol 76(3):174–187. 10.1016/j.biopsycho.2007.07.006 17888560 10.1016/j.biopsycho.2007.07.006

[CR33] Haggard P (2008) Human volition: towards a neuroscience of will. Nat Rev Neurosci 9(12):934–946. 10.1038/nrn2497 19020512 10.1038/nrn2497

[CR34] Haggard P (2019) The neurocognitive bases of human volition. Annu Rev Psychol 70:9–28. 10.1146/annurev-psych-010418-103348. (**Epub 2018 Aug 20 PMID: 30125134**) 30125134 10.1146/annurev-psych-010418-103348

[CR35] Haggard P, Eimer M (1999) On the relation between brain potentials and the awareness of voluntary movements. Exp Brain Res 126:128–133 10333013 10.1007/s002210050722

[CR36] Haggard P, Parés- Pujolràs E (2021) What are the main stages in the neural processes that produce actions? In: Free Will. Uri Maoz and Walter Sinnott- Armstrong (Eds) Oxford University Press. 10.1093/oso/9780197572153.003.0016

[CR37] Hassannejad NA, Liljenström H (2015) A cortical network model of cognitive and emotional influences in human decision making. BioSystems 13:128–141 10.1016/j.biosystems.2015.07.004 26184761

[CR38] Haynes JD (2011) Beyond Libet: Long-term prediction of free choices from neuroimaging signals. In: Sinnott-Armstrong W, Nadel L (eds) Conscious will and responsibility. Oxford University Press, Oxford, pp 85–96

[CR39] Hebb DO (1949) The organization of behavior; a neuropsychological theory. Wiley

[CR40] Hrvoj-Mihic B, Semendeferi K (2019) Neurodevelopmental disorders of the prefrontal cortex in an evolutionary context. Prog Brain Res 250:109–127. 10.1016/bs.pbr.2019.05.003. (**Epub 2019 Jul 12 PMID: 31703898**) 31703898 10.1016/bs.pbr.2019.05.003

[CR41] Hutt A, Beim Graben P (2017) Sequences by metastable atractors: interweaving dynamical systems and experimental data. Front Appl Math Stat 3:1–14

[CR42] Jenison RL (2014) Directional influence between the human amygdala and orbitofrontal cortex at the time of decision-making. PLoS ONE 9(10):e109689. 10.1371/journal.pone.0109689 25333929 10.1371/journal.pone.0109689 PMC4204819

[CR43] Kang C, Li Y, Novak D, Zhang Y, Zhou Q, Hu Y (2020) Brain networks of maintenance, inhibition and disinhibition during working memory. IEEE Trans Neural Syst Rehabil Eng 28(7):1518–1527. 10.1109/TNSRE.2020.2997827 32634090 10.1109/TNSRE.2020.2997827

[CR44] Katayama N, Nakagawa A, Umeda S, Terasawa Y, Kurata C, Tabuchi H, Kikuchi T, Mimura M (2019) Frontopolar cortex activation associated with pessimistic future-thinking in adults with major depressive disorder. Neuroimage Clin 23:101877. 10.1016/j.nicl.2019.101877 31170685 10.1016/j.nicl.2019.101877 PMC6551553

[CR45] Keller I, Heckhausen H (1990) Readiness potentials preceding spontaneous motor acts: voluntary vs. involuntary control. Electroencephalogr Clin Neurophysiol 76:351–361 1699728 10.1016/0013-4694(90)90036-j

[CR46] Kornhuber HH, Deecke L (1965) Hirnpotentialandeerungen Bei Wilkurbewegungen Und Passiv Bewegungen Des Menschen: Bereitschaftspotential Und Reafferente Potentiale. Pflugers Archiv Fur Gesamte Psychologie 284:1–17 14341490

[CR47] Lee VK, Harris LT (2013) How social cognition can inform social decision making. Front Neurosci 7:259. 10.3389/fnins.2013.00259.PMID:24399928;PMCID:PMC3872305 24399928 10.3389/fnins.2013.00259 PMC3872305

[CR48] Libet B (2004) Mind time. Harvard University Press, London

[CR49] Libet B, Wright EW, Gleason CA (1982) Readiness potentials preceding unrestricted “spontaneous” vs. pre-planned voluntary acts. Electroencephalogr Clin Neurophysiol 54:322–335 6179759 10.1016/0013-4694(82)90181-x

[CR50] Libet B, Gleason CA, Wright EW, Pearl DK (1983) Time of conscious intention to act in relation to onset of cerebral activity (readiness-potential) – the unconscious initiation of a freely voluntary act. Brain 106(3):623–642. 10.1093/brain/106.3.623 6640273 10.1093/brain/106.3.623

[CR51] Liljenström H (1991) Modeling the dynamics of olfactory cortex using simplified network units and realistic architecture. Int J Neural Syst 02(01n02):1–15. 10.1142/S0129065791000029

[CR52] Liljenström H (1995) Autonomous learning with complex dynamics. Int J Intell Syst 10(1):119–153

[CR53] Liljenström H (2011) Intention and attention in consciousness dynamics and evolution. J Cosmol 14:4848–4858

[CR54] Liljenström H (2018) Intentionality as a driving force. J Consc Stud 25(1–2):206–229

[CR55] Liljenström H (2022) Consciousness, decision-making, and volition – freedom beyond chance and necessity. Theory Biosci 141(2):125–140 34046848 10.1007/s12064-021-00346-6 PMC9184456

[CR56] Liljenström H, Hasselmo ME (1995) Cholinergic modulation of cortical oscillatory dynamics. J Neurophysiol 74(1):288–297. 10.1152/jn.1995.74.1.288. (**PMID: 7472331**) 7472331 10.1152/jn.1995.74.1.288

[CR57] Liljenstrom H, Wu XB (1995) Noise-enhanced performance in a cortical associative memory model. Int J Neural Syst 6(1):19–29 7670670 10.1142/s0129065795000032

[CR58] Maoz U, Yaffe G, Koch C, Mudrik L (2019) Neural precursors of decisions that matter—an ERP study of deliberate and arbitrary choice. Elife 8:e39787. 10.7554/eLife.39787 31642807 10.7554/eLife.39787 PMC6809608

[CR59] Matilla-García M, Morales I, Rodríguez JM, Ruiz Marín M (2021) Selection of embedding dimension and delay time in phase space reconstruction via symbolic dynamics. Entropy (basel) 23(2):221. 10.3390/e23020221.PMID:33670103;PMCID:PMC7916852 33670103 10.3390/e23020221 PMC7916852

[CR60] Medalla M, Barbas H (2010) Anterior cingulate synapses in prefrontal areas 10 and 46 suggest differential influence in cognitive control. J Neurosci off J Soc Neurosci 30(48):16068–16081. 10.1523/JNEUROSCI.1773-10.2010 10.1523/JNEUROSCI.1773-10.2010 PMC3064955 21123554

[CR61] Medalla M, Barbas H (2012) The anterior cingulate cortex may enhance inhibition of lateral prefrontal cortex via m2 cholinergic receptors at dual synaptic sites. J Neurosci 32(44):15611–15625. 10.1523/JNEUROSCI.2339-12.2012.PMID:23115196;PMCID:PMC3523186 23115196 10.1523/JNEUROSCI.2339-12.2012 PMC3523186

[CR62] Mele A (2009) Effective intentions: the power of conscius will. Oxford University Press, New York

[CR63] Mele A (2019) Free will and neuroscience: decision times and the point of no return. In: Free Will, Causality, and Neuroscience (B. Feltz, M. Missal and A. Sims, Eds) Brill

[CR64] Miller EK, Cohen JD (2001) An integrative theory of prefrontal cortex function. Annu Rev Neurosci 24:167–202. 10.1146/annurev.neuro.24.1.167 11283309 10.1146/annurev.neuro.24.1.167

[CR65] Miller J, Schwarz W (2014) Brain signals do not demonstrate unconscious decision making: an interpretation based on graded conscious awareness. Conscious Cogn 24:12–21. 10.1016/j.concog.2013.12.004 24394375 10.1016/j.concog.2013.12.004

[CR66] Mushiake H, Saito N, Sakamoto K, Itoyama Y, Tanji J (2006) Activity in the lateral prefrontal cortex reflects multiple steps of future events in action plans. Neuron 50(4):631–641. 10.1016/j.neuron.2006.03.045. (**PMID: 16701212**) 16701212 10.1016/j.neuron.2006.03.045

[CR67] Nachev P, Wydell H, O’neill K, Husain M, Kennard C (2007) The role of the pre-supplementary motor area in the control of action. Neuroimage 36(3–3):155–163 10.1016/j.neuroimage.2007.03.034 PMC2648723 17499162

[CR68] Nachev P, Kennard C, Husain M (2008) Functional role of the supplementary and pre-supplementary motor areas. Nat Rev Neurosci 9(11):856–869. 10.1038/nrn2478 18843271 10.1038/nrn2478

[CR69] Oberauer K (2019) Working memory and attention—a conceptual analysis and review. J Cogn 2(1):36. 10.5334/joc.58.PMID:31517246;PMCID:PMC6688548 31517246 10.5334/joc.58 PMC6688548

[CR70] Okuda J, Fujii T, Ohtake H, Tsukiura T, Yamadori A, Frith CD, Burgess PW (2007) Differential involvement of regions of rostral prefrontal cortex (Brodmann area 10) in time- and event-based prospective memory. Int J Psychophysiol 64(3):233–246. 10.1016/j.ijpsycho.2006.09.009. (**Epub 2006 Nov 28 PMID: 17126435**) 17126435 10.1016/j.ijpsycho.2006.09.009

[CR71] Palomero-Gallagher N, Mohlberg H, Zilles K, Vogt B (2008) Cytology and receptor architecture of human anterior cingulate cortex. J Comp Neurol 508(6):906–926. 10.1002/cne.21684.PMID:18404667;PMCID:PMC2678551 18404667 10.1002/cne.21684 PMC2678551

[CR72] Parés-Pujolràs E, Haggard P (2021) What are intentions and intentional actions? In: Free will. (Ed) Uri Maozn, and Walter Sinnott- Armstrong, Oxford University Press. © Oxford University Press. 10.1093/oso/9780197572153.003.0021

[CR73] Peng K, Steele SC, Becerra L, Borsook D (2018) Brodmann area 10: Collating, integrating and high level processing of nociception and pain. Prog Neurobiol 161:1–22. 10.1016/j.pneurobio.2017.11.004 29199137 10.1016/j.pneurobio.2017.11.004 PMC5826795

[CR74] Petrides M (2005) Lateral prefrontal cortex: architectonic and functional organization. Philos Trans R Soc Lond B Biol Sci 360(1456):781–795. 10.1098/rstb.2005.1631 15937012 10.1098/rstb.2005.1631 PMC1569489

[CR75] Pribram KH, Mishkin M, Rosvold HE, Kaplan SJ (1952) Effects on delayed-response performance of lesions of dorsolateral and ventromedial frontal cortex of baboons. J Comp Physiol Psychol 45(6):565–575. 10.1037/h0061240 13000029 10.1037/h0061240

[CR76] Ramnani N, Owen AM (2004) Anterior prefrontal cortex: insights into function from anatomy and neuroimaging. Nat Rev Neurosci 5(3):184–194. 10.1038/nrn1343. (**PMID: 14976518**) 14976518 10.1038/nrn1343

[CR77] Rossi AF, Pessoa L, Desimone R, Ungerleider LG (2009) The prefrontal cortex and the executive control of attention. Exp Brain Res 192(3):489–497. 10.1007/s00221-008-1642-z 19030851 10.1007/s00221-008-1642-z PMC2752881

[CR78] Schoemann M, Scherbaum S (2020) From high- to one-dimensional dynamics of decision making: testing simplifications in attractor models. Cogn Process 21:303–313. 10.1007/s10339-020-00953-z 32016686 10.1007/s10339-020-00953-z PMC7203584

[CR79] Schurger A, Hu P, Pak J, Roskies AL (2021) What is the readiness potential? Trends Cogn Sci 25(7):558–570. 10.1016/j.tics.2021.04.001 33931306 10.1016/j.tics.2021.04.001 PMC8192467

[CR80] Seghezzi S, Zapparoli L (2020) Predicting the sensory consequences of self-generated actions: pre-supplementary motor area as supra-modal hub in the sense of agency experience. Brain Sci 10(11):825. 10.3390/brainsci10110825 33171715 10.3390/brainsci10110825 PMC7694977

[CR81] Shipp S (2005) The importance of being agranular: a comparative account of visual and motor cortex. Philos Trans R Soc Lond B Biol Sci 360(1456):797–814. 10.1098/rstb.2005.1630 15937013 10.1098/rstb.2005.1630 PMC1569485

[CR82] Soon CS, Brass M, Heinzev HJ, Haynes JD (2008) Unconscious determinants of free decisions in the human brain. Nat Neurosci 11(5):543–545. 10.1038/nn.2112 18408715 10.1038/nn.2112

[CR83] Stalnaker TA, Cooch NK, Schoenbaum G (2015) What the orbitofrontal cortex does not do. Nat Neurosci 18(5):620–627. 10.1038/nn.3982.PMID:25919962;PMCID:PMC5541252 25919962 10.1038/nn.3982 PMC5541252

[CR84] Sussillo D, Abbott LF (2009) Generating coherent patterns of activity from chaotic neural networks. Neuron 63(4):544–557. 10.1016/j.neuron.2009.07.018 19709635 10.1016/j.neuron.2009.07.018 PMC2756108

[CR85] Takens F (1981) Detecting strange attractors in turbulence. In: Rand DA, and Young LS (ed) Dynamical systems and turbulence, Lecture Notes in Mathematics, vol 898. Springer-Verlag. pp 366–381

[CR86] Tanji J, Hoshi E (2008) Role of the lateral prefrontal cortex in executive behavioral control. Physiol Rev 88(1):37–57. 10.1152/physrev.00014.2007. (**PMID: 18195082**) 18195082 10.1152/physrev.00014.2007

[CR87] Trevena JA, Miller J (2002) Cortical movement preparation before and after a conscious decision to move. Conscious Cogn 11:162–190 12191935 10.1006/ccog.2002.0548

[CR88] Unsworth N, Engle RW (2007) The nature of individual differences in working memory capacity: active maintenance in primary memory and controlled search from secondary memory. Psychol Rev 114(1):104–132. 10.1037/0033-295x.114.1.104 17227183 10.1037/0033-295X.114.1.104

[CR89] Unsworth N, Spillers GJ (2010) Working memory capacity: attention control, secondary memory, or both? a direct test of the dual-component model. J Mem Lang 62(4):392–406. 10.1016/j.jml.2010.02.001

[CR90] Van Inwagen P (2017) Thinking about free will. Cambridge University Press

[CR91] Wu X, Liljenström H (1994) Regulating the nonlinear dynamics of olfactory cortex. Netw Comput Neural Syst 5:47–60

